# Supplementary material for: Development and prospective external validation of a tool to predict poor recovery at 9 months after acute ankle sprain in UK emergency departments: the SPRAINED prognostic model
Source: BMJ Open. 2018 Nov 5;8(11):e022802. doi: 10.1136/bmjopen-2018-022802 (PMC6231561; doi:10.1136/bmjopen-2018-022802)
Supplement: Supplementary data [file bmjopen-2018-022802supp001.pdf]

Supplemental Table 1. List of candidate predictor variables from the ED presentation.

ED Proforma

1. Date of birth
2. Sex (male / female / no response)
3. Date of ED visit
4. Date of injury
5. Location of pain
6. Anterior Drawer Test (Positive / Painful / Negative / no response)
7. Talar Tilt Test (Positive / Painful / Negative / no response)
8. Proximal Fibular tender (Positive / Painful / Negative / no response)
9. Weight bearing ability (Full / Partial / None / no response)
10. X-ray (yes / no / no response)
11. Crutches (yes / no/ no response)
12. Trial Reason for not entering trial (ankle fracture / other recent fracture / contraindication to intervention / poor skin viability / > 7 days since injury / other)
13. Additional information (if other)
14. Recruiting Centre
15. Date of Trial Clinic
16. Days since injury

Supplemental Table 2. List of candidate predictor variables from the Baseline assessment datasets.

Identifier variables

1. Trial Centre
2. Patient's ID
3. Date of assessment
4. Randomisation Group
5. Treatment Received
6. Calendar code
7. Calendar colour
8. Indicator of Pilot Study phase (I / II / main trial)
9. Response at baseline (yes / no)

Background Information Form

10. Age (years)
11. Sex (male / female)
12. Ethnic Group (White / Black-Caribbean / Black-African / Black-Other / Indian / Pakistani / Bangladeshi / Chinese / Other)
13. Ethnic Group details (if other)
14. First language (English / Other European / Gujarati / Hindi / Punjabi / Urdu / Bengali / Other)
15. First language additional info (if other)
16. Able to answer English questions? (yes / no)
17. Current employment status (full-time / part-time / unemployed)
18. Employment category (paid / unpaid)
19. Hours employed (Less than 10 / 10-25 / 25-40 / More than 40 hours per week)
20. Type of employment (Unskilled manual / skilled manual / unskilled non-manual / skilled non-manual / professional / other / decline d to answer)
21. Description of employment (if professional)
22. Description of employment (if other)
23. Occupation if not employed (retired / not looking for work / unable to work / looking for work / full time student / other)
24. Description of unemployment (if other)
25. Education (CSE / O-Level or GCSE / A-level / degree / higher degree / other)
26. Description of level of education (if other)
27. Time on feet (most of the day / > 4 hours a day / < 4 hours a day / Not much time, mostly sitting)
28. Time driving (most of the day / > 4 hours a day / < 4 hours a day / just to & from work / don't drive)
29. Current medications (since ankle injury / prior to injury / no / no answer)
30. Practice of physical activities (11 questions) (> once a week / < once a week / never)
41. Other physical activity (if other)
42. Height (cm)
43. Weight (kg)
44. Pain before injury (yes/no)
45. When had previous pain (exercise or heavy activities / exercise and daily activities / constantly / other)
46. Description of when had previous pain (if other)
47. Frequency of previous pain (never / monthly / weekly / daily / always)
48. Previous instability (yes / no)
49. Severity of instability (mild / moderate / severe)
50. Frequency of instability (rarely / sometimes / frequently / always)
51. Previous injury (yes / no)
52. 3+ previous injuries (yes / no)
53. Previous injury <1 year ago (yes / no)
54. Recurrent sprain – yes to all 3 above (yes / no)
55. A&E attendance previously (yes / no)
56. How present injury occurred (during sport / at work / at home / outside in public place / other)
57. Description of how present injury occurred
58. Maximum weight bearable (kg)

Baseline questionnaire

59. FAOS components (42 questions)
101. Pain at rest VAS (0-100)
102. Pain bearing weight VAS (0-100)
103. FAOS Baseline symptoms (subscale)
104. FAOS Baseline pain (subscale)
105. FAOS Baseline function ADL (subscale)
106. FAOS Baseline function sport (subscale)
107. FAOS Baseline QoL (subscale)
108. FLP components (13 questions)
121. WORK components (10 questions)
131. FLP score
132. WORK score
133. 1998 SF-12 components (12 questions)
145. 1998 SF-12 physical score
146. 1998 SF-12 mental score
147. Baseline EQ-5D components (5 questions)
152. Baseline EQ-5D score
153. General level of health today (better / same / worse than the past 6 months)
154. VAS health today (0-100)

Note: Imputed scores of validated scales with specific rules for handling missing data imputation (such as FAOS, SF-12 and EQ-5D) are also present in the CAST dataset, but were not described here.

Supplemental Table 3. Pre-selected baseline and 4-weeks candidate predictor variables.

Type	Variable name	Categories / units	Missing values	
Baseline				
Binary	Gender	Male, female	0	0%
	Previous pain	Yes, No	26	4%
	Recurrent sprain	Yes, No	12	2%
Categorical	Employment status	No, Part time, Full time	0	0%
	Education	CSE, GCSE, A Level, Degree, Higher Degree	20	3%
	Anterior Drawer Test	Positive, Painful, Negative, No response	396	68%
	Talar Tilt Test	Positive, Painful, Negative, No response	403	69%
	Proximal Fibular tender ligament test	Positive, Painful, Negative, No response	378	65%
	Able to bear weight	Full / partial / none	322	55%
	Treatment group	Tubular bandage, Below knee cast, Aircast brace, Bledsoe boot	0	0%
	Leisure time physical activity	None, <1 weekly, >1 weekly	7	1%
	Walking 2 miles or more	None, <1 weekly, >1 weekly	24	4%
	Previous instability	None, Mild, Moderate, Severe	27	5%
	Previous instability frequency	Never, Rarely, Sometimes, Frequently, Always	29	5%
	Injury presentation	During sport, at work, at home, outside in public	34	6%
	Ankle/foot Swelling[1]	Never, Rarely, Sometimes, Often, Always	18	3%
	Ankle/foot Grinding/clicking[1]	Never, Rarely, Sometimes, Often, Always	18	3%
	Ankle/foot catching/locking[1]	Never, Rarely, Sometimes, Often, Always	18	3%
	Ankle ROM plantar flexion[1]	Never, Rarely, Sometimes, Often, Always	18	3%
	Ankle ROM plantar dorsiflexion[1]	Never, Rarely, Sometimes, Often, Always	18	3%
	Pain at night (on bed) [1]	None, Mild, Moderate, Severe, Extreme	18	3%
	Difficulty with squatting[1]	None, Mild, Moderate, Severe, Extreme	29	5%
	Difficulty with running[1]	None, Mild, Moderate, Severe, Extreme	31	5%
	Difficulty with jumping[1]	None, Mild, Moderate, Severe, Extreme	31	5%
	Difficulty with twisting/pivoting[1]	None, Mild, Moderate, Severe, Extreme	26	4%
Continuous	Time since injury	0-7 days	321	55%
	Age	Years[2]	0	0%
	Body mass index[3]	kg/m^2	19	3%
	Maximum weight bearable	kg	5	1%
	Pain when resting	Visual analogue scale (0-100)	4	1%
	Pain when bearing weight	Visual analogue scale (0-100)	9	2%
	SF-12 mental component	Score (0-100)	5	1%
4-weeks				
Binary	Repeat injury to the same ankle	Yes, No	118	20%
	Returned to ED due to repeated injury	Yes, No	120	21%
Categorical	Returned to usual sports/activities	No, partially, fully	121	21%
	Ankle/foot Swelling	Never, Rarely, Sometimes, Often, Always	102	17%
	Ankle/foot Grinding/clicking	Never, Rarely, Sometimes, Often, Always	102	17%
	Ankle/foot catching/locking	Never, Rarely, Sometimes, Often, Always	103	18%
	Ankle ROM plantar flexion	Never, Rarely, Sometimes, Often, Always	102	17%
	Ankle ROM plantar dorsiflexion	Never, Rarely, Sometimes, Often, Always	102	17%
	Pain at night	None, Mild, Moderate, Severe, Extreme	101	17%
	Difficulty with squatting	None, Mild, Moderate, Severe, Extreme	101	17%
	Difficulty with running	None, Mild, Moderate, Severe, Extreme	135	22%
	Difficulty with jumping	None, Mild, Moderate, Severe, Extreme	137	23%
	Difficulty with twisting/pivoting	None, Mild, Moderate, Severe, Extreme	131	22%
Continuous	Pain at weight bearing	0-100	196	34%

Supplemental Table 4. Reasons for exclusion of baseline and 4-weeks candidate predictors before statistical modelling.

**Predictor**	**Reason for exclusion**
Baseline	
Anterior Drawer Test	≥ 60% missing values, consensus agreement
Talar Tilt Test	≥ 60% missing values, consensus agreement
Proximal Fibular tender ligament test	≥ 60% missing values, consensus agreement
Ankle/foot Swelling	One or more cells with too few cases when cross-tabulated with the outcomes, regardless of re-categorisation
Difficulty with running	Highly correlated with “Difficulty with jumping” and “Difficulty with twisting/pivoting”. Composite variable used instead.
Difficulty with jumping	Highly correlated with “Difficulty with running” and “Difficulty with twisting/pivoting”. Composite variable used instead.
Difficulty with twisting/pivoting	Highly correlated with “Difficulty with running” and “Difficulty with jumping”. Composite variable used instead.
Previous instability	Highly correlated with “Previous instability frequency”
Previous instability frequency	“Perfect prediction” during missing data multiple imputation
Difficulty with running/jumping/twisting	“Perfect prediction” during missing data multiple imputation
4 weeks follow-up	
Returned to ED due to repeated injury	One or more cells with too few cases when cross tabulated with the outcomes
Difficulty with running	Highly correlated with “Difficulty with jumping” and “Difficulty with twisting/pivoting”. Composite variable used instead.
Difficulty with jumping	Highly correlated with “Difficulty with running” and “Difficulty with twisting/pivoting”. Composite variable used instead.
Difficulty with twisting/pivoting	Highly correlated with “Difficulty with running” and “Difficulty with jumping”. Composite variable used instead.
Ankle ROM plantar dorsiflexion	“Perfect prediction” during missing data multiple imputation

Note: Perfect prediction occurs whenever there is a level of a categorical explanatory variable for which the observed values of the outcome are all 1 (or all 0); it is often resolved by discarding the observations corresponding to offending covariate patterns or the independent variables perfectly predicting outcomes during estimation.

Supplemental Table 5. SPRAINED baseline and updated prognostic models' equations.

Baseline SPRAINED prognostic model:

Y = -3.68 + (0.02 × age) + (0.02 × BMI) + (0.01 × pain when resting) + (0.01 × pain when bearing weight) + (0.61 if days from injury to assessment > 2) – (0.56 if able to bear any weight on the injured ankle) + (0.84 if the injury is a recurrent sprain)

Updated SPRAINED prognostic model (baseline + 4-weeks predictors):

Y = -4.4 + (0.01 × age) + (0.02 × BMI) + (0.01 × pain when resting) + (0.01 × pain when bearing weight) + (0.59 if days from injury to assessment > 2) – (0.68 if able to bear any weight on the injured ankle) + (0.99 if the injury is a recurrent sprain) + (0.02 × pain when bearing weight 4 weeks after injury)

The log odds (*Y*) estimated with these equations must be converted into a probability (*P*), which can be done by applying the following equation:

$$P = 1/(1 + \exp(-Y))$$

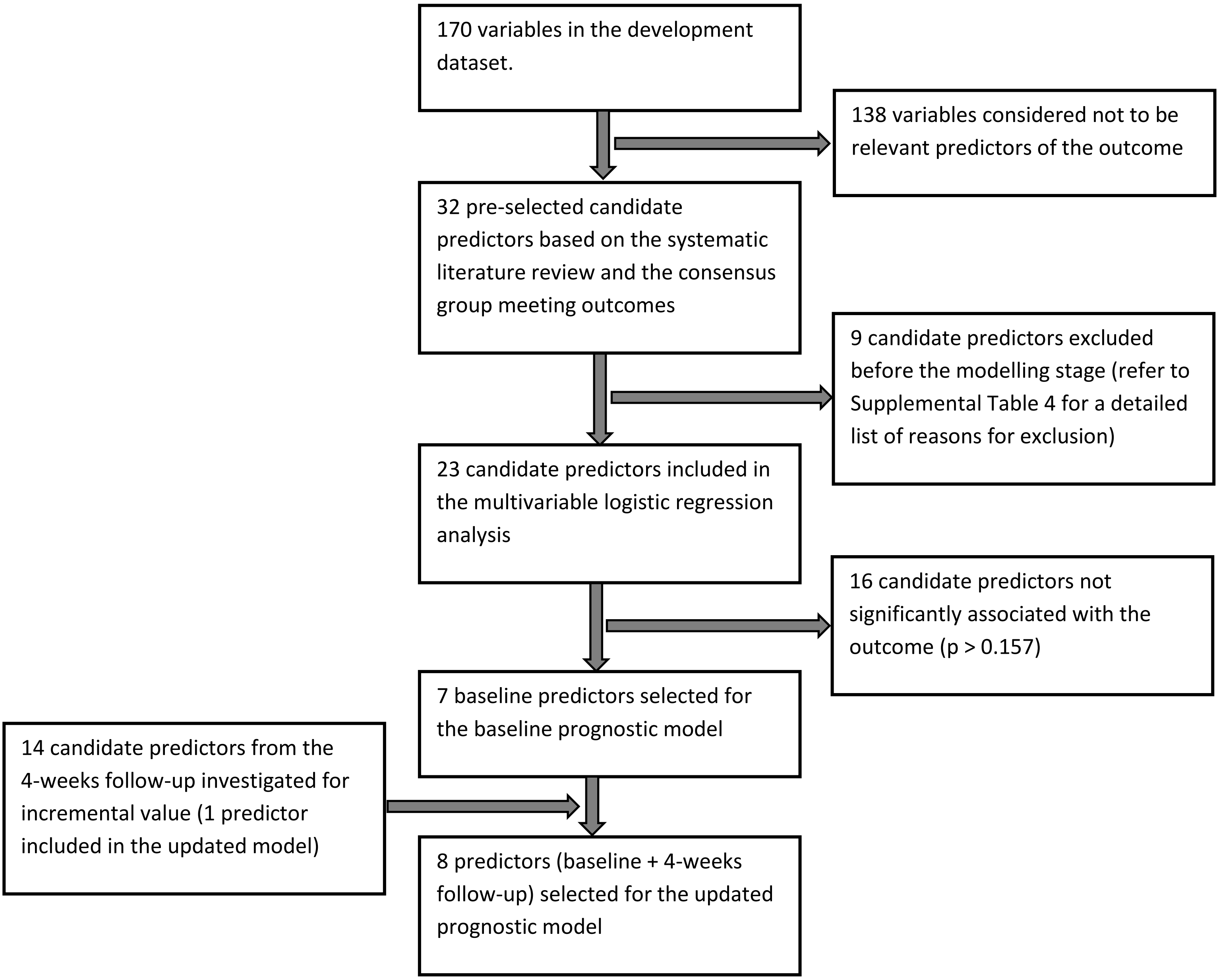

Supplemental Figure 1. Summary of the selection process for predictors included in the SPRAINED prognostic model.
