# Supplementary material for: Development and prospective external validation of a tool to predict poor recovery at 9 months after acute ankle sprain in UK emergency departments: the SPRAINED prognostic model
Source: BMJ Open. 2018 Nov 5;8(11):e022802. doi: 10.1136/bmjopen-2018-022802 (PMC6231561; doi:10.1136/bmjopen-2018-022802)
Supplement: Author's manuscript [file bmjopen-2018-022802.draft_revisions.pdf]

BMJ Open is committed to open peer review. As part of this commitment we make the peer review history of every article we publish publicly available.

When an article is published we post the peer reviewers' comments and the authors' responses online. We also post the versions of the paper that were used during peer review. These are the versions that the peer review comments apply to.

The versions of the paper that follow are the versions that were submitted during the peer review process. They are not the versions of record or the final published versions. They should not be cited or distributed as the published version of this manuscript.

BMJ Open is an open access journal and the full, final, typeset and author-corrected version of record of the manuscript is available on our site with no access controls, subscription charges or pay-per-view fees (http://bmjopen.bmj.com).

If you have any questions on BMJ Open's open peer review process please email editorial.bmjopen@bmj.com

BMJ Open

DEVELOPMENT AND EXTERNAL VALIDATION OF A TOOL TO PREDICT POOR RECOVERY AT NINE MONTHS AFTER ACUTE ANKLE SPRAIN: THE SPRAINED PROGNOSTIC MODEL

Journal:	*BMJ Open*
Manuscript ID	bmjopen-2018-022802
Article Type:	Research
Date Submitted by the Author:	09-Mar-2018

Complete List of Authors:	Schlussel, Michael; Centre for Statistics in Medicine, NDORMS<br>Keene, David; University of Oxford, Centre for Rehabilitation Research, Nuffield Departments of Orthopaedic Rheumatology and Musculoskeletal Sciences<br>Collins, Gary; University of Oxford, Centre for Statistics in Medicine<br>Bostock, Jennifer; Quality and Outcomes of Person-Centred Care Policy Research Unit<br>Byrne, Christopher; University of Plymouth, School of Health Professions, Faculty of Health and Human Sciences<br>Goodacre, Steve; University of Sheffield, Medical Care Research Unit<br>Gwilym, Stephen; University of Oxford, Oxford Trauma, Nuffield Departments of Orthopaedic Rheumatology and Musculoskeletal Sciences<br>Hagan, Daryl; University of Oxford, Centre for Rehabilitation Research, Nuffield Departments of Orthopaedic Rheumatology and Musculoskeletal Sciences<br>Haywood, Kirstie; University of Warwick, Warwick Research in Nursing, Division of Health Sciences, Warwick Medical School<br>Thompson, Jacqueline; University of Oxford, Centre for Rehabilitation Research, Nuffield Departments of Orthopaedic Rheumatology and Musculoskeletal Sciences<br>Williams , Mark ; Oxford Brookes University, Sport and Health Sciences<br>Lamb, Sarah; University of Oxford, Ndorms

Keywords:	prognosis, clinical prediction rule, logistic model, ankle injuries, sprains and strains

SCHOLARONE™
Manuscripts

DEVELOPMENT AND EXTERNAL VALIDATION OF A TOOL TO PREDICT POOR RECOVERY AT NINE MONTHS AFTER ACUTE ANKLE SPRAIN: THE SPRAINED PROGNOSTIC MODEL

Michael M Schlüssel [1*], David J Keene [2], Gary S Collins[1], Jennifer Bostock[3], Christopher Byrne[4], Steve Goodacre[5], Steve Gwilym[6], Daryl A Hagan[2], Kirstie Haywood[7], Jacqueline Thompson[2], Mark A Williams[8], Sarah E Lamb [1,2] for the SPRAINED study team.

[1] Centre for Statistics in Medicine, Nuffield Departments of Orthopaedic Rheumatology and Musculoskeletal Sciences, University of Oxford, Oxford, United Kingdom

[2] Centre for Rehabilitation Research, Nuffield Departments of Orthopaedic Rheumatology and Musculoskeletal Sciences, University of Oxford, Oxford, United Kingdom

[3] Patient and Public Involvement representative, Quality and Outcomes of Person-Centred, Care Policy Research Unit, Canterbury, Kent, UK

[4] Faculty of Health and Human Sciences, University of Plymouth, Plymouth, United Kingdom

[5] School of Health and Related Research, University of Sheffield, Sheffield, United Kingdom

[6] Oxford Trauma, Nuffield Departments of Orthopaedic Rheumatology and Musculoskeletal Sciences, University of Oxford, Oxford, United Kingdom

[7] Warwick Research in Nursing, Division of Health Sciences, Warwick Medical School, University of Warwick, Coventry, United Kingdom

[8] Department of Sport, Health Sciences and Social Work, Oxford Brookes University, Oxford, United Kingdom

* Corresponding author

Address for correspondence:

Dr Michael Maia Schlüssel
Centre for Statistics in Medicine
Botnar Research Centre, Old Road
OX3 7LD
Oxford, United Kingdom
Email michael.schlussel@csm.ox.ac.uk
Telephone: +44 01865 737916

Word count: 3,479 words

For peer review only - http://bmjopen.bmj.com/site/about/guidelines.xhtml

ABSTRACT

OBJECTIVES: We aimed to develop and externally validate a prognostic model to estimate the risk of poor recovery after ankle sprain. **SETTING AND PARTICIPANTS:** Model development used secondary data analysis from 584 participants in the CAST multicentre randomised clinical trial. External validation used data from 682 participants recruited in 10 emergency departments across the UK for the SPRAINED prospective observational cohort. **OUTCOME AND ANALYSIS:** Poor recovery was defined as the presence of pain, functional difficulty or lack of confidence in the ankle at 9-months after injury. Twenty-three baseline candidate predictors were included together in a multivariable logistic regression model to identify the best predictors of poor recovery. Relationships between continuous variables and the outcome were modelled using fractional polynomials. Regression parameters were combined over 50 imputed datasets using Rubin's rule. To minimise over-fitting, regression coefficients were multiplied by a heuristic shrinkage factor and the intercept re-estimated. Incremental value of candidate predictors assessed at 4-weeks after injury was explored using decision curve analysis and the baseline model updated. The final models included predictors selected based on the Akaike Information Criterion ($p<0.157$). Model performance was assessed by calibration and discrimination. **RESULTS:** Outcome rate was lower in SPRAINED (6.7%) than in CAST (19.9%). Mean age (29.9 and 33.6 years), BMI (26.3 and 27.1 kg/m^2), pain when resting (37.8 and 38.5 points) or bearing weight on the ankle (75.4 and 71.3 points) were similar in both datasets. Seven baseline and one 4-weeks predictors were included in the models. The baseline model had fair discriminatory ability (c-statistic 0.72; 95%CI: 0.66-0.79) but poor calibration. The updated model presented better discrimination (c-statistic 0.78; 95%CI: 0.72-0.84), but equivalent calibration. **CONCLUSIONS:** The models include predictors easy to assess clinically and show benefit when compared to not using any model. **Registry number:** ISRCTN12726986.

Keywords: prognosis, clinical prediction rule, logistic model, ankle injuries, sprains and strains

For peer review only - http://bmjopen.bmj.com/site/about/guidelines.xhtml

STRENGTHS AND LIMITATIONS OF THIS STUDY

- This is the first study to develop and externally validate a tool to predict poor recovery after ankle sprain, including a wide range of clinically relevant candidate predictors.
- Despite containing information on the outcomes of interest and numerous prognostic variables, the development dataset was not originally acquired to build a prognostic model.
- The number of events in the development dataset was relatively small compared to the number of candidate predictors examined.
- Yet, the prognostic models were developed using robust statistical methods, adjusted for overfitting and reported according to the most recent relevant guidelines available.
- Generalisability of findings is enhanced by the multi-centre characteristic of the datasets used in the development and external validation of the models.

For peer review only

INTRODUCTION

Ankle sprains are one of the most common musculoskeletal injuries, representing up to 5% of all emergency department (ED) attendances in the UK.**[1]** Despite heterogeneity in sampling frame (e.g. restricted to elite athletes or excluding older people), inception, and follow-up time points, studies have indicated that approximately 30% of people have persistent problems one year after ankle sprain.**[2, 3]** In a large multi-centre randomised clinical trial conducted in the UK, a similar proportion (30%) of participants had poor outcome at 9 months.**[4]** Other studies indicate a recovery plateau at around 9 months, and residual disability after this point to be persistent.**[5]**

In the acute phase after sprain, physical examination of the ankle is often limited by swelling and pain. Predicting prognosis at this stage is uncertain and is based on clinical judgement. In a recent systematic literature review, a few prognostic models aimed at patients with ankle sprains were identified, but these were of limited generalisability due to selective patient populations or explored a limited number of candidate prognostic factors.**[6]**

The aim of our study was to develop and externally validate the SPRAINED (Synthesising a clinical Prognostic Rule for Ankle Injuries in the Emergency Department) prognostic model, to identify people at risk of poor recovery at nine months after acute ankle sprain.

METHODS

Study populations and data collection

Data from the Collaborative Ankle Support Trial (CAST), were used to develop the prognostic model.**[7]** CAST was a pragmatic multicentre randomised controlled trial on the effectiveness of different mechanical ankle supports compared with a double-layer tubular compression bandage for managing severe ankle sprains. The trial sample comprised 584 participants aged 16 years or older, with an ankle sprain of grade 2 or 3, attending eight EDs in the UK between April 2003 and July 2005, within 7 days after their injury, and were not able to fully bear weight on the injured ankle at baseline. Further data was collected at 4 and 12 weeks, and 9 months after randomisation. The CAST methods are available elsewhere.**[7]**

To assess the model's performance in an external population, the SPRAINED prospective observational cohort was recruited. Participants were aged 16 years or above, with acute

For peer review only - http://bmjopen.bmj.com/site/about/guidelines.xhtml

ankle sprains of any grade, attending 10 NHS EDs across England, within 7 days after their injury. Patients were excluded if they presented with an ankle fracture (except flake fractures < 2mm) or any other recent (< 3 months) lower limb fracture. Participants were not randomised, nor did they receive any interventions other than usual care at each site. The study recruited 682 participants between July 2015 and March 2016. Data collection covered clinical and socio-demographic information collected at ED presentation (baseline), with follow-up assessments at 4 weeks, 4 and 9 months after the initial injury, either by self-reported paper-based forms sent back to the study office by postal mail, electronic questionnaires, or telephone interviews. The SPRAINED questionnaires included all variables selected as predictors in the model and the components of the outcome of interest. Ethics approval was from the National Research Ethics Committee (REC) (London - Chelsea), REC number 15/LO/0538, on 10th April 2015. The study protocol was registered on 30th April 2015; registry number ISRCTN12726986.

Definition of outcome

A prognostic model was developed to predict 'poor recovery' at 9 months after an acute ankle sprain. Poor recovery was defined as the presence of pain, lack of confidence in the ankle (persistent feeling of giving way) or functional difficulty.**[8-9]** The presence of these symptoms was assessed by patient-reported responses given to specific items (P1, Q3 and Q4) of the Foot and Ankle Outcome Score (FAOS).**[10]** Participants who answered one or more of these questions with any of the two most extreme response options ("daily" or "always" P1; "severely" or "extremely" for Q3 or Q4) were considered to have poor outcome.

Baseline candidate predictors

Thirty-two baseline candidate predictors were considered plausible predictors of poor outcome and pre-selected from a pool of 170 variables available in the CAST dataset. This initial selection was made internally by the research team, taking into account the results from our systematic literature review **[6]** and the conclusions from a consensus group meeting convened for the SPRAINED study, which included clinicians, medical researchers, statisticians and PPI representatives. The 32 candidate predictors included socio-demographic information (e.g. age, sex, body mass index (BMI), education, employment status); pre-injury quality of life, mobility and lifestyle indicators (e.g. engagement in sports

For peer review only - http://bmjopen.bmj.com/site/about/guidelines.xhtml

activities); clinical data on injury presentation; baseline (post-injury) mobility levels, pain and weight-bearing status (**Supplemental file 1).**

At this stage, variables were excluded or combined before statistical modelling if they had 60% or more of missing information; displayed high collinearity ($r \geq 0.8$) with another candidate predictor; presented empty or low cell counts ($n < 5$) when tabulated against the outcome; were the offending variable causing perfect prediction during the multiple imputation process (**Supplemental file 2**).

Sample size considerations

It is widely recommended that the dataset used to develop a prognostic tool should contain a minimum of 5-10 outcome events per variable (EPV) included as a predictor in the model.**[11-16]** After the exclusion of nine baseline candidate predictors for the reasons described above, 23 variables from baseline remained as candidate predictors. However, some of these predictors were categorical variables with more than two levels, so we ended with 35 candidate parameters, meaning the EPV ratio was approximately three.

As to the best of our knowledge this was the first study aiming to develop prediction models to assess the risk of poor recovery after an acute ankle sprain, we opted for relaxing the EPV rule in favour of including more potentially important predictors. Nevertheless, we adopted several strategies to minimise bias and overfitting, as described below.

Descriptive analysis

Baseline and 4-week follow-up characteristics of the CAST and SPRAINED participants were summarised using means, standard deviations (SDs) and ranges for continuous variables, or counts and percentages for categorical variables. Inspection of extreme values (outliers) took place to confirm whether they were clinically plausible and visual assessment of data distribution for continuous predictors in both datasets was conducted. No formal statistical tests were performed to compare the values between the studies.

Prognostic model development

Using logistic regression, we developed the prognostic model to predict the probability of poor recovery. We performed multiple imputation using chained equations (MICE) **[17]** to handle missing data, with 50 imputed datasets created. Continuous variables were kept as

For peer review only - http://bmjopen.bmj.com/site/about/guidelines.xhtml

For peer review only

continuous to avoid loss of prognostic information,**[18]** and the shape of their relationship with the outcome studied and modelled with nonlinear functions such as fractional polynomials (FPs) where appropriate.**[19]** As several continuous variables were included in the models, we used the multivariable fractional polynomial (MFP) algorithm.**[20, 21]**

Multiple imputation and fractional polynomials were combined using the *mfpmi* function in Stata.**[22]** The estimated regression parameters (coefficients and variances) were combined over the 50 imputed datasets using Rubin's rule.**[23, 24]** After identifying the best transformation terms for continuous variables in the full multivariable models with all candidate predictors, the final model included predictors (and respective transformations, where applicable) selected based on the Akaike Information Criterion (equivalent to a p-value < 0.157).**[25]** To adjust for over-fitting, due to small EPV, we multiplied all regression coefficients by the heuristic shrinkage factor,**[26]** then re-estimated the intercept. All model assumptions were checked and differences between incomplete and imputed datasets inspected. Imputed data from all 584 participants were included in all analyses.

Incremental value analysis and model update

In addition to the baseline predictors, 14 additional variables from the CAST 4-weeks follow-up questionnaire were also selected as potential predictors that could increase the model's prognostic ability (**Supplemental file 1**). First, all additional 4-weeks candidate predictors were included together in the final baseline model and only those achieving a p-value < 0.157 were considered for inclusion in the updated model (i.e. a model including baseline and 4-weeks predictors). Finally, the updated model was compared with the original baseline model using decision curve analysis (DCA) plots to determine whether the inclusion of additional predictors reflected in increased net benefit.**[27, 28]**

External validation: Model performance

We assessed the model performance in the prospectively collected SPRAINED cohort. Missing data in the SPRAINED cohort was handled using MICE, creating 50 imputed datasets. Performance was evaluated by assessing calibration and discrimination. Calibration is the agreement between observed and predicted probabilities of poor outcome. Calibration was assessed graphically using calibration plots, with observed risks plotted on the *y*-axis against predicted risks on the *x*-axis.**[29, 30]** The calibration plot was created by regressing the

For peer review only - http://bmjopen.bmj.com/site/about/guidelines.xhtml

outcome on the predicted probability using a locally weighted scatter plot smoother (lowess). The calibration plot was also supplemented with estimates of the calibration slope and intercept. Models with perfect calibration will have a calibration slope of 1 and intercept 0 (i.e., prediction lying on the 45° line). Calibration plots followed recommendations of overlaying calibration curves from each imputed data set.**[31]** Discrimination reflects the ability of the model to distinguish between participants who did and did not experience an event during the study period. Discrimination was assessed using the c-statistic, where a value of 0.5 represents chance and 1 represents perfect discrimination.**[32]** Finally, to estimate the benefit of using the developed models, patients were ranked according to their estimated risks. These were used to calculate the number of people per 1000 identified as being at high risk according to selected thresholds and how many of these went on to present the outcomes compared with not using the model. Individual probabilities of developing the outcomes were estimated by applying the developed prognostic models to each participant in the SPRAINED imputed datasets. We assessed the performance of both the baseline and updated models using imputed data from all 682 participants.

Patient involvement

A PPI representative was involved in the study from the beginning, providing advice on key aspects of the study design, including the definition of the research question, choice of the outcome and selection of relevant candidate predictors during the consensus group meeting. They will be consulted for the public dissemination of any product arriving from this research.

Reporting

We followed the TRIPOD Statement for the reporting of our study.**[33]**

RESULTS

Baseline characteristics for the CAST (development) and SPRAINED (validation) cohorts are summarised in **Table 1**. On average, participants were slightly older in SPRAINED than in CAST (33.6 vs. 29.9 years, respectively). Participants in SPRAINED had an average BMI within the overweight category (27.1 kg/m2), likewise those in CAST (26.3 kg/m2). The mean pain scores when resting (38.5 points) or bearing weight on the ankle (71.3 points) of SPRAINED

For peer review only - http://bmjopen.bmj.com/site/about/guidelines.xhtml

participants were also similar to those observed for CAST participants (37.8 and 75.4 points, respectively). Differently from CAST, in SPRAINED about half of participants were female (52.1%), the majority presented to an ED within 2 days from injury for assessment (90.0%) and were able to bear some weight on their injured ankles (73.6%) (**Table 1**).

For peer review only

For peer review only - http://bmjopen.bmj.com/site/about/guidelines.xhtml

Table 1. Baseline characteristics of the participants in the CAST trial and SPRAINED prospective observational cohort.

Variable	CAST Trial		SPRAINED Cohort	
	**Mean (SD)**	**Min - Max**	**Mean (SD)**	**Min - Max**
Age (years)	29.88 (10.77)	16 – 72	33.62 (13.38)	16 – 89
Height (m)	1.73 (0.98)	1.47 – 2.01	1.72 (1.02)	1.50 – 2.01
Weight (kg)	78.56 (15.44)	39.92 – 133.36	80.44 (18.13)	44.50 – 180
Body mass index (kg/m^2)	26.34 (5.19)	16.07 – 53.77	27.08 (5.70)	17.31 – 64.30
Pain when resting (score)	37.75 (23.49)	0 – 100	38.50 (22.50)	0 – 100
Pain when bearing weight (score)	75.42 (19.61)	0 – 100	71.30 (21.00)	0 – 100
	**Frequency**	**%**	**Frequency**	**%**
Sex				
Male	337	57.71	327	47.95
Female	247	42.29	355	52.05
Days from injury to assessment				
0-2	118	44.87	614	90.03
3 or more	145	55.13	68	9.97
Able to bear weight at Baseline assessment				
No	446	77.03	179	26.44
Yes	133	22.97	498	73.56
Recurrent sprain				
No	517	90.38	583	91.38
Yes	55	9.62	55	8.62
Current employment				
None	132	22.60	161	23.68
Part time	92	15.75	92	13.53
Full time	360	61.64	427	62.79
Injury mechanism				
At home	99	18.00	144	21.56
Practicing sports	203	36.91	230	34.43
At work	79	14.36	91	13.62
Outside, in public	169	30.73	203	30.39

Table 2 shows the rates of poor recovery in the CAST trial and SPRAINED cohort datasets, as well as the number of its component symptoms, at 9 months after injury. There was a lower rate of poor recovery in the SPRAINED cohort (6.7%) than observed in the CAST trial (19.9%), but the percentage of missing data for the outcome was similar in both studies (22.7% and 24.7%, respectively).

For peer review only

For peer review only - http://bmjopen.bmj.com/site/about/guidelines.xhtml

Table 2. Outcome and respective symptoms components rates and proportion of missing data in the CAST trial and SPRAINED prospective observational cohort.

	Pain	Lack of confidence	Instability	Poor recovery	Missing data	TOTAL[1]
CAST	84 (14.4%)	42 (7.2%)	67 (11.5%)	116 (19.9%)	144 (24.7%)	584
SPRAINED	3 (0.4%)	23 (3.4%)	37 (5.4%)	46 (6.7%)	155 (22.7%)	682

Note: Poor recovery defined as the presence of one or more of the following symptoms: pain, lack of confidence or instability/difficulty with the ankle.

Table 3 displays the summary of the final multivariable models (predictor's coefficients, respective 95% confidence intervals and p-values). Seven of the 23 baseline candidate predictors were selected for inclusion in the baseline model: age, BMI, pain when resting, pain when bearing weight, days from injury to assessment, ability to bear weight and whether or not the injury was a recurrent sprain. Although not statistically significant in the final model according to AIC (p<0.157), BMI was kept as a baseline predictor given its clinical importance. This decision also prevented another round of predictors selection, which could increase over-fitting. The best fit for all continuous predictors were linear transformations (mean subtractions) and were later incorporated into the model by updating the intercept accordingly (**Supplemental file 3**).

For peer review only

Table 3. Summary of the final baseline and updated (baseline plus 4-weeks predictors) logistic regression models and respective shrunk coefficients and intercepts.

Predictors	Baseline model					Updated model (baseline plus 4-weeks predictors)				
	Coefficient	95% CI		p	Shrunk coefficient	Coefficient	95% CI		P	Shrunk coefficient
Age	0.027	0.006	0.048	0.014	0.019	0.018	-0.005	0.040	0.127	0.015
BMI	0.031	-0.014	0.076	0.178	0.022	0.025	-0.022	0.072	0.292	0.021
Pain when resting	0.016	0.005	0.027	0.005	0.011	0.010	-0.002	0.022	0.107	0.008
Pain when bearing weight	0.019	0.004	0.035	0.016	0.014	0.014	-0.002	0.030	0.092	0.012
Pain when bearing weight 4 wks. after injury	-	-	-	-	-	0.022	0.012	0.032	< 0.001	0.018
Days from injury to assessment (reference 0-2)										
3 or more	0.854	0.068	1.640	0.034	0.605	0.702	-0.117	1.520	0.092	0.591
Able to bear weight at Baseline (reference No)										
Yes	-0.792	-1.376	-0.207	0.008	-0.561	-0.802	-1.412	-0.192	0.010	-0.676
Recurrent sprain (reference No)										
Yes	1.180	0.417	1.944	0.003	0.836	1.170	0.386	1.953	0.004	0.985
Intercept	-1.580	-2.152	-1.008	< 0.001	-1.363	-1.543	-2.128	-0.958	< 0.001	-1. 420

95% CI: 95% confidence interval

Linear terms selected by the MFP for continuous predictors: Age – 29.88; BMI – 26.32; Pain when resting – 37.75; Pain when bearing weight – 75.40; Pain when bearing weight at 4 weeks after injury – 36.23.

For peer review only - http://bmjopen.bmj.com/site/about/guidelines.xhtml

Only pain when bearing weight on the sprained ankle at 4 weeks after injury was included in the updated model (baseline plus 4-week predictors) (**Table 3**). By inspecting the DCA plots shown in **Figure 1** it is possible to see a clear net benefit gain over the entire range of thresholds when using the updated prognostic model in comparison to the baseline model or considering all patients (or no patient) at risk of having poor recovery after an acute ankle sprain.

Shrinkage suggested both prognostic models (baseline and updated) had predictor-outcome associations that were too large. The heuristic shrinkage factor for the coefficients of the predictors in the baseline prognostic model was 0.71. For the updated version (baseline plus 4-weeks predictors), the estimated heuristic shrinkage factor was 0.84. The shrunk coefficients and intercepts for the final models are presented in **Table 3**.

Overall, discrimination of the baseline model was fair, with a c-statistic of 0.72 (95%CI: 0.66 to 0.79). Calibration of the baseline prognostic model in the external validation dataset was poor though, as can be evidenced by inspecting the calibration plot with overlaid calibration lines from the 50 imputed datasets (**Figure 2**). The calibration slope was 1.13 (95%CI: 0.76 to 1.5) and the calibration intercept was -0.71 (95%CI: -0.98 to -0.44). The updated model (baseline plus 4-weeks predictors) presented better discriminatory ability in the SPRAINED dataset than the baseline model (c-statistic = 0.78; 95%CI: 0.72 to 0.84), but equivalent calibration, with an intercept closer to zero (-0.51; 95%CI: -0.78 to -0.24) and slope slightly further from one (1.17; 95%CI: 0.86 to 1.48).

Table 4 shows how many of 1000 people would be identified as being at high risk (based on thresholds of 5, 10, 15, and 20%) using the developed prognostic models, and how many of these would actually present poor recovery 9 months after an acute ankle sprain. There seems to be little difference between the baseline and updated models, with both identifying similar numbers of patients who would experience a poor outcome after an acute ankle sprain. However, less patients are deemed at high risk by using the updated model for (less false positives) across all thresholds of predicted probability suggesting that reassessing the patients at 4 weeks after the injury might be beneficial to a more accurate prediction of their probability of poor outcome. Using any of the models is clearly beneficial, when compared to not using any model (i.e. considering all patients – or no patients – as high risk of developing poor outcome).

For peer review only - http://bmjopen.bmj.com/site/about/guidelines.xhtml

Table 4. Models performance (numbers at risk and outcomes identified) at varying risk thresholds for 1000 patients.

Selected thresholds	Number of patients at risk		Number of events	
	High risk	Low risk	Identified	Not identified
Consider all high risk	1000	0	85	0
Predicted probability as per baseline model				
≥5%	971	39	85	0
≥10%	797	203	74	11
≥15%	543	457	63	22
≥20%	351	649	52	33
Predicted probability as per updated model (baseline plus 4-weeks predictors)				
≥5%	882	118	85	0
≥10%	517	483	71	14
≥15%	358	642	56	29
≥20%	259	741	41	44

DISCUSSION

We developed a prognostic model to predict a composite outcome representing the presence of at least one of the following symptoms at 9 months after an acute ankle sprain: pain, functional difficulty or lack of confidence in the ankle. The model presented fair discriminatory ability in a prospectively collected external validation cohort, but poor calibration. Including an additional variable collected at 4 weeks after the injury (pain when bearing weight on the injured ankle) improved the discriminatory ability and calibration of the model. The models included predictors that are easily collected and provided reasonable predictions of poor recovery for patients with acute ankle sprain.

To the best of our knowledge, this is the first study to develop and externally validate a prognostic model to predict a clinically relevant outcome in people with acute ankle sprains exploring a wide range of clinically plausible candidate predictors. We used robust statistical methods to select the predictors and assess the model´s performance in a large external prospective cohort. Generalisability of the findings are enhanced by the multi-centre data from the CAST and SPRAINED cohorts that represented a range of district general and major trauma centres. The observational cohort we prospectively recruited for SPRAINED is representative of patients presenting to EDs in the UK. We followed the most recent and complete guidelines available on the reporting of prognostic model development,**[33]** and applied recommended methods to minimise overfitting. For example, continuous variables, whenever possible, were kept as continuous to avoid loss of information. Nonlinear relationships were investigated using the best variables transformations found by

For peer review only

293 multivariable fractional polynomials. The study included an internal correction for model optimism (shrinkage of regression coefficients and re-estimation of intercepts) as well as a prospective external validation phase. The amount of missing data in the external validation dataset, which is commonplace in studies of this nature, was considerably smaller than that observed in the development dataset. Finally, we performed missing data imputation to produce a set of 50 complete datasets and enable robust analyses.

299 Limitations of the SPRAINED study are acknowledged. Firstly, data used to develop the prognostic models were from a prior randomised controlled trial (CAST), so were not originally intended to fulfil this aim. However, the CAST cohort did represent the best dataset available, with information on the symptoms and clinical events of interest, and a wide range of the candidate prognostic variables considered to have predictive ability. Secondly, the CAST dataset used to develop the prognostic model was relatively small compared to the number of candidate predictors.**[11-16]** As previously highlighted, the low EPV observed for the two developed models might have contributed to the optimism found for both and, therefore, to the poor calibration on the external validation dataset. Thirdly, the amount of missing data observed in the development dataset. Because of that, a number of candidate predictors were omitted before the process of data imputation, to avoid instability of the imputation models. Therefore, some important predictors could have conceivably been missed in the development phase of the SPRAINED study. Finally, the rates of poor outcome in the SPRAINED cohort were lower than in the CAST trial and those reported in previous systematic reviews.**[2, 3]** These variations in poor outcome rates highlight the potential issue of different sampling frames.

315 The prognostic models have the potential to assist clinicians deciding whether to refer a patient a review clinic and to offer some reassurance to people not referred that they are likely to be on a positive recovery trajectory. Improved predictive performance of the models with the addition of information on pain on weight bearing at 4 weeks indicates that re-assessment of prognosis after the acute phase is worth consideration. Given some limitations in predictive performance of the SPRAINED prognostic models, we suggest that their value would be in assisting estimating the probability of a poor outcome, rather than being a decision rule in isolation. If implemented in clinical practice, it should be noted that there is a degree of uncertainty in the calculated risk of poor outcome when using the

For peer review only - http://bmjopen.bmj.com/site/about/guidelines.xhtml

SPRAINED models. This uncertainty in estimation could lead to over or under referral of patients to review clinics or treatment such as physiotherapy, and highlights the caution required when using the calculated individual risks when counselling patients about their prognosis. We recommend that further research should aim to evaluate the impact of using the SPRAINED prognostic models on clinical practice and patient outcomes and to assess the acceptability and uptake of use by emergency department clinicians.

In conclusion, the SPRAINED prognostic models performed reasonably and despite some miscalibration show benefit in identifying patients at high risk of poor outcome after an acute ankle sprain. The models may assist clinical-decision making when assessing and advising people with ankle sprains in the ED setting and when deciding on on-going management. The models benefit from using predictors that are simple to obtain during routine clinical assessment.

DATA SHARING

All data requests should be submitted to the corresponding author for consideration. Access to anonymised data may be granted following review. Exclusive use will be retained until the publication of major outputs.

FUNDING

The SPRAINED study was funded by the National Institute of Health Research (NIHR) Health Technology Assessment programme (project number 13/19/06). Supported by the NIHR Biomedical Research Centre, Oxford, and the NIHR Fellowship programme (Dr David Keene, PDF-2016-09-056). Sarah Lamb receives funding from the National Institute for Health Research (NIHR) Collaboration for Leadership in Applied Health Research and Care Oxford at Oxford Health NHS Foundation Trust. The views and opinions expressed therein are those of the authors and do not necessarily reflect those of the Health Technology Assessment programme, NIHR, NHS or the Department of Health.

COMPETING INTERESTS

None declared.

For peer review only - http://bmjopen.bmj.com/site/about/guidelines.xhtml

AUTHOR'S CONTRIBUTIONS

MMS analysed and interpreted the data, and led the writing of the manuscript. DJK had substantial contribution in data acquisition, analysis and interpretation. GSC had substantial contribution in the study conception and design, data analysis and interpretation. JB had substantial contribution in the study conception and design. CB had substantial contribution in the data acquisition. SG had substantial contribution in the study conception and design. SG had substantial contribution in the study conception and design. DAH had substantial contribution in the data acquisition. KH had substantial contribution in the study conception and design. JT had substantial contribution in the data acquisition. MAW had substantial contribution in the study conception and design, data acquisition. SEL was responsible for the study conception and design, had substantial contribution in data interpretation. All authors revised and approved the final version of the manuscript.

ACKNOWLEDGEMENTS

The SPRAINED study team members are: Sarah E Lamb (Chief Investigator); David J Keene (Study Lead); Gary S Collins, Mark Williams, Steve Goodacre, Matthew Cooke, Steve Gwilym, Phil Hormbrey, David Wilson, Jennifer Bostock (Co-investigators); Daryl Hagan (Study administrator); Damian Haywood (Senior study manager); Jacqueline Thompson, Christopher Byrne (Research Physiotherapists); Michael Maia Schlüssel (Study statistician); Philip Hormbrey, Susan Dorrian, Steve Goodacre, Victoria Stacey, Tim Coats, Sarah Wilson, Jason Kendall, David Clarke, Antoanela Colda, Deborah Mayne (Principal Investigators and their clinical and research teams at collaborating recruitment centres); Kirstie Haywood (consultation and senior facilitation of the consensus meeting). We would like to thanks Prof. Richard Riley, Prof. Kevin Mackway-Jones and Prof. Suzanne McDonough from the Independent Study Steering Committee for their invaluable comments, support and advise throughout the study. We also acknowledge the contributions of Vicki Barber and the staff members at Oxford Clinical Trials Research Unit and Centre for Rehabilitation Research for their support in delivering the SPRAINED study; the members of the consensus group meeting, including the PPI representatives; and all those who have in some way contributed to the conception, conduction and reporting of the SPRAINED study.

For peer review only

REFERENCES

1. Wilson RW, Gansneder BM. Measures of functional limitation as predictors of disablement in athletes with acute ankle sprains. *J Orthop Sports Phys Ther* 2000;30:528-35.

2. van Rijn RM, van Os AG, Bernsen RM, et al. What is the clinical course of acute ankle sprains? A systematic literature review. *Am J Med* 2008;121:324-31.

3. Polzer H, Kanz KG, Prall WC, et al. Diagnosis and treatment of acute ankle injuries: development of an evidence-based algorithm. *Orthop Rev* (Pavia) 2012;4:e5.

4. Cooke MW, Marsh JL, Clark M, et al. Treatment of severe ankle sprain: a pragmatic randomised controlled trial comparing the clinical effectiveness and cost-effectiveness of three types of mechanical ankle support with tubular bandage. The CAST trial. Health technology assessment 2009;13:iii, ix-x, 1-121.

5. Verhagen RA, de Keizer G, van Dijk CN. Long-term follow-up of inversion trauma of the ankle. *Arch Orthop Trauma Surg* 1995;114:92-6.

6. Thompson JY, Byrne C, Williams MA, et al. Prognostic factors for recovery following acute lateral ankle ligament sprain: a systematic review. *BMC Musculoskelet Disord*. 2017;18:421.

7. Lamb SE, Marsh JL, Hutton JL, et al. Mechanical supports for acute, severe ankle sprain: a pragmatic, multicentre, randomised controlled trial. *Lancet* 2009;373:575-81.

8. van Rijn RM, Willemsen SP, Verhagen AP, et al. Explanatory variables for adult patients' self-reported recovery after acute lateral ankle sprain. *Phys Ther* 2011;91:77-84.

9. Wikstrom EA, Hubbard-Turner T, McKeon PO. Understanding and treating lateral ankle sprains and their consequences: a constraints-based approach. *Sports Med* 2013;43:385-93.

10. Roos EM, Brandsson S, Karlsson J. Validation of the foot and ankle outcome score for ankle ligament reconstruction. *Foot Ankle Int* 2001;22:788-94.

11. Harrell Jr FE, Lee KL, Califf RM, et al. Regression modelling strategies for improved prognostic prediction. *Stat Med* 1984;3:143-52.

For peer review only - http://bmjopen.bmj.com/site/about/guidelines.xhtml

12. Harrell Jr FE, Lee KL, Mark DB. Multivariable prognostic models: issues in developing models, evaluating assumptions and adequacy, and measuring and reducing errors. *Stat Med* 1996;15:361-87.

13. Peduzzi P, Concato J, Kemper E, et al. A simulation study of the number of events per variable in logistic regression analysis. *J Clin Epidemiol* 1996;49:1373-9.

14. Moons KG, de Groot JA, Bouwmeester W, et al. Critical Appraisal and Data Extraction for Systematic Reviews of Prediction Modelling Studies: The CHARMS Checklist. *PLoS med* 2014;11:e1001744.

15. Pavlou M, Ambler G, Seaman SR, et al. How to develop a more accurate risk prediction model when there are few events. *BMJ* 2015;351:h3868.

16. Vittinghoff E, McCulloch CE. Relaxing the rule of ten events per variable in logistic and Cox regression. *Am J Epidemiol* 2007;165:710-8.

17. White IR, Royston P, Wood AM. Multiple imputation using chained equations: Issues and guidance for practice. *Stat Med* 2011;30:377-99.

18. Collins GS, Ogundimu EO, Cook JA, et al. Quantifying the impact of different approaches for handling continuous predictors on the performance of a prognostic model. *Stat Med* 2016;35:4124-35.

19. Royston P, Altman DG. Regression Using Fractional Polynomials of Continuous Covariates: Parsimonious Parametric Modelling. *J R Stat Soc Ser C Appl Stat* 1994;43:429-67.

20. Royston P, Sauerbrei W. MFP: Multivariable Model-Building with Fractional Polynomials. In: Multivariable Model-Building: John Wiley & Sons, Ltd; 2008:115-50.

21. Sauerbrei W, Royston P, Binder H. Selection of important variables and determination of functional form for continuous predictors in multivariable model building. *Stat Med* 2007;26:5512-28.

22. Morris TP, White IR, Carpenter JR, et al. Combining fractional polynomial model building with multiple imputation. *Stat Med* 2015;34:3298-317.

23. Marshall A, Altman DG, Holder RL, et al. Combining estimates of interest in prognostic modelling studies after multiple imputation: current practice and guidelines. *BMC Med Res Methodol* 2009;9:57.

For peer review only

For peer review only - http://bmjopen.bmj.com/site/about/guidelines.xhtml

24. Rubin DB. Multiple Imputation for Nonresponse in Surveys: Wiley; 2004.

25. Atkinson AC. A note on the generalized information criterion for choice of a model. *Biometrika* 1980;67:413-8.

26. van Houwelingen JC, LeCessie S. Predictive value of statistical models. *Stat Med* 1990;9:1303-25.

27. Vickers AJ, Elkin EB. Decision curve analysis: a novel method for evaluating prediction models. *Med Decis Making* 2006;26:565-74.

28. Vickers AJ, Van Calster B, Steyerberg EW. Net benefit approaches to the evaluation of prediction models, molecular markers, and diagnostic tests. *BMJ* 2016;352:i6.

29. Austin PC, Steyerberg EW. Graphical assessment of internal and external calibration of logistic regression models by using loess smoothers. *Stat Med* 2014;33:517-35.

30. Wood AM, Royston P, White IR. The estimation and use of predictions for the assessment of model performance using large samples with multiply imputed data. *Biom J* 2015; 10.1002/bimj.201400004.

31. Janssen KJ, Moons KG, Kalkman CJ, et al. Updating methods improved the performance of a clinical prediction model in new patients. *J Clin Epidemiol* 2008;61:76-86.

32. Steyerberg EW, Vickers AJ, Cook NR, et al. Assessing the performance of prediction models: a framework for traditional and novel measures. *Epidemiology* 2010;21:128-38.

33. Collins GS, Reitsma JB, Altman DG, et al. Transparent reporting of a multivariable prediction model for individual prognosis or diagnosis (TRIPOD): the TRIPOD statement. *Ann Intern Med* 2015;162:55-63.

FIGURE LEGENDS

Figure 1. Decision curve analysis for the baseline and updated (baseline plus 4-weeks predictors) prognostic models.

Figure 2. Calibration plots for the baseline (left) and updated (right) SPRAINED prognostic models, overlaying calibration lines derived from the analyses of 50 imputed datasets.

For peer review only - http://bmjopen.bmj.com/site/about/guidelines.xhtml

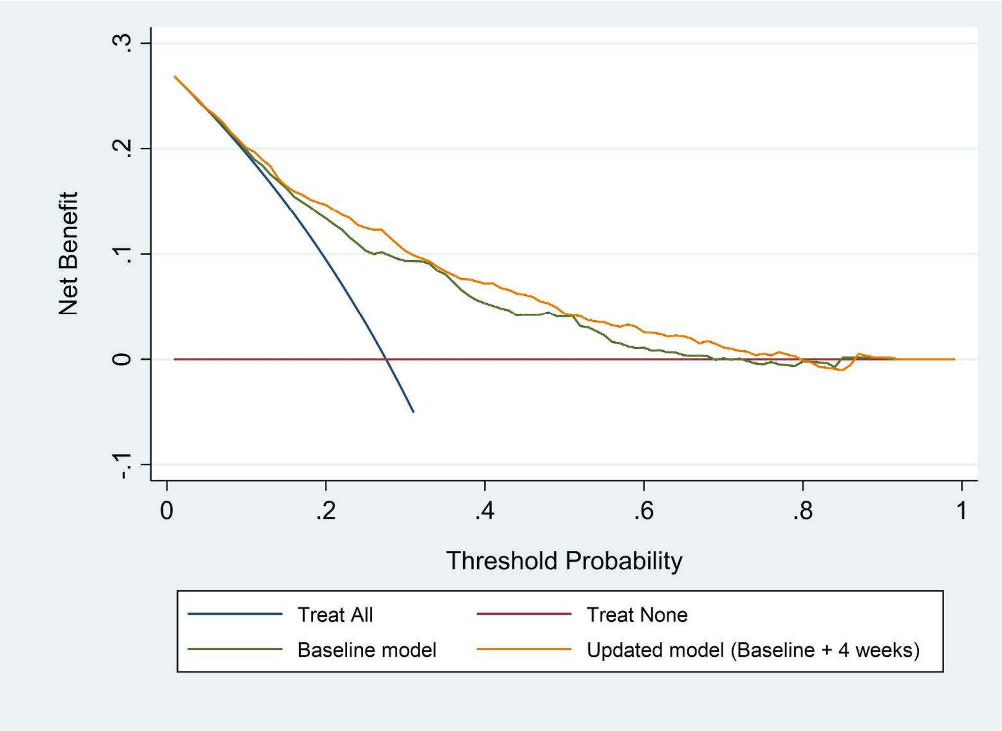

Figure 1. Decision curve analysis for the baseline and updated (baseline plus 4-weeks predictors) prognostic models.

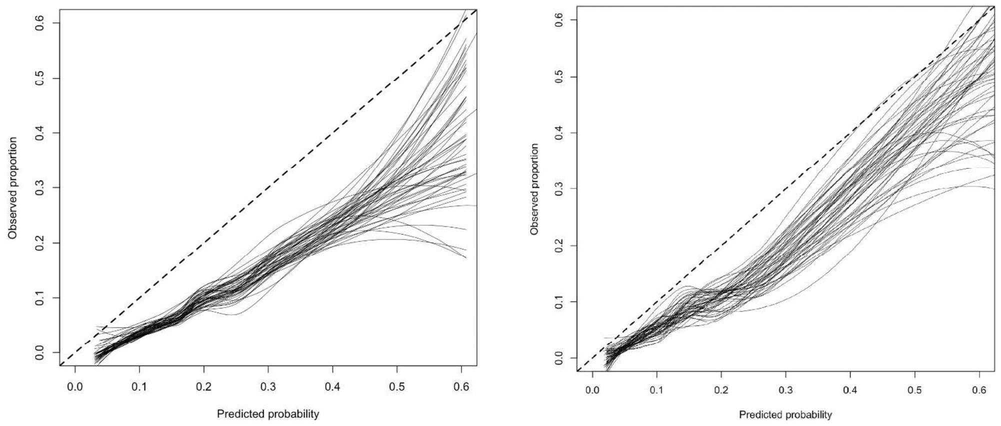

Figure 2. Calibration plots for the baseline (left) and updated (right) SPRAINED prognostic models, overlaying calibration lines derived from the analyses of 50 imputed datasets.

For peer review only - http://bmjopen.bmj.com/site/about/guidelines.xhtml

Supplemental Table 1. Pre-selected baseline and 4-weeks candidate predictor variables.

Type	Variable name	Categories / units	Missing values	
Baseline				
Binary	Gender	Male, female	0	0%
	Previous pain	Yes, No	26	4%
	Recurrent sprain	Yes, No	12	2%
Categorical	Employment status	No, Part time, Full time	0	0%
	Education	CSE, GCSE, A Level, Degree, Higher Degree	20	3%
	Anterior Drawer Test	Positive, Painful, Negative, No response	396	68%
	Talar Tilt Test	Positive, Painful, Negative, No response	403	69%
	Proximal Fibular tender ligament test	Positive, Painful, Negative, No response	378	65%
	Able to bear weight	Full / partial / none	322	55%
	Treatment group	Tubular bandage, Below knee cast, Aircast brace, Bledsoe boot	0	0%
	Leisure time physical activity	None, <1 weekly, >1 weekly	7	1%
	Walking 2 miles or more	None, <1 weekly, >1 weekly	24	4%
	Previous instability	None, Mild, Moderate, Severe	27	5%
	Previous instability frequency	Never, Rarely, Sometimes, Frequently, Always	29	5%
	Injury presentation	During sport, at work, at home, outside in public	34	6%
	Ankle/foot Swelling[(1)]	Never, Rarely, Sometimes, Often, Always	18	3%
	Ankle/foot Grinding/clicking[(1)]	Never, Rarely, Sometimes, Often, Always	18	3%
	Ankle/foot catching/locking[(1)]	Never, Rarely, Sometimes, Often, Always	18	3%
	Ankle ROM plantar flexion[(1)]	Never, Rarely, Sometimes, Often, Always	18	3%
	Ankle ROM plantar dorsiflexion[(1)]	Never, Rarely, Sometimes, Often, Always	18	3%
	Pain at night (on bed)[(1)]	None, Mild, Moderate, Severe, Extreme	18	3%
	Difficulty with squatting[(1)]	None, Mild, Moderate, Severe, Extreme	29	5%
	Difficulty with running[(1)]	None, Mild, Moderate, Severe, Extreme	31	5%
	Difficulty with jumping[(1)]	None, Mild, Moderate, Severe, Extreme	31	5%
	Difficulty with twisting/pivoting[(1)]	None, Mild, Moderate, Severe, Extreme	26	4%
Continuous	Time since injury	0-7 days	321	55%
	Age	Years[(2)]	0	0%
	Body mass index[(3)]	kg/m^2	19	3%
	Maximum weight bearable	kg	5	1%
	Pain when resting	Visual analogue scale (0-100)	4	1%
	Pain when bearing weight	Visual analogue scale (0-100)	9	2%
	SF-12 mental component	Score (0-100)	5	1%
4-weeks				
Binary	Repeat injury to the same ankle	Yes, No	118	20%
	Returned to ED due to repeated injury	Yes, No	120	21%
Categorical	Returned to usual sports/activities	No, partially, fully	121	21%
	Ankle/foot Swelling	Never, Rarely, Sometimes, Often, Always	102	17%
	Ankle/foot Grinding/clicking	Never, Rarely, Sometimes, Often, Always	102	17%
	Ankle/foot catching/locking	Never, Rarely, Sometimes, Often, Always	103	18%
	Ankle ROM plantar flexion	Never, Rarely, Sometimes, Often, Always	102	17%
	Ankle ROM plantar dorsiflexion	Never, Rarely, Sometimes, Often, Always	102	17%
	Pain at night	None, Mild, Moderate, Severe, Extreme	101	17%
	Difficulty with squatting	None, Mild, Moderate, Severe, Extreme	101	17%
	Difficulty with running	None, Mild, Moderate, Severe, Extreme	135	22%
	Difficulty with jumping	None, Mild, Moderate, Severe, Extreme	137	23%
	Difficulty with twisting/pivoting	None, Mild, Moderate, Severe, Extreme	131	22%
Continuous	Pain at weight bearing	0-100	196	34%

For peer review only

Supplemental Table 2. Reasons for exclusion of baseline and 4-weeks candidate predictors before statistical modelling.

Predictor	Reason for exclusion
Baseline	
Anterior Drawer Test	≥ 60% missing values, consensus agreement
Talar Tilt Test	≥ 60% missing values, consensus agreement
Proximal Fibular tender ligament test	≥ 60% missing values, consensus agreement
Ankle/foot Swelling	One or more cells with too few cases when cross-tabulated with the outcomes, regardless of re-categorisation
Difficulty with running	Highly correlated with “Difficulty with jumping” and “Difficulty with twisting/pivoting”. Composite variable used instead.
Difficulty with jumping	Highly correlated with “Difficulty with running” and “Difficulty with twisting/pivoting”. Composite variable used instead.
Difficulty with twisting/pivoting	Highly correlated with “Difficulty with running” and “Difficulty with jumping”. Composite variable used instead.
Previous instability	Highly correlated with “Previous instability frequency”
Previous instability frequency	“Perfect prediction” during missing data multiple imputation
Difficulty with running/jumping/twisting	“Perfect prediction” during missing data multiple imputation
4 weeks follow-up	
Returned to ED due to repeated injury	One or more cells with too few cases when cross tabulated with the outcomes
Difficulty with running	Highly correlated with “Difficulty with jumping” and “Difficulty with twisting/pivoting”. Composite variable used instead.
Difficulty with jumping	Highly correlated with “Difficulty with running” and “Difficulty with twisting/pivoting”. Composite variable used instead.
Difficulty with twisting/pivoting	Highly correlated with “Difficulty with running” and “Difficulty with jumping”. Composite variable used instead.
Ankle ROM plantar dorsiflexion	“Perfect prediction” during missing data multiple imputation

Note: Perfect prediction occurs whenever there is a level of a categorical explanatory variable for which the observed values of the outcome are all 1 (or all 0); it is often resolved by discarding the observations corresponding to offending covariate patterns or the independent variables perfectly predicting outcomes during estimation.

For peer review only

Supplemental Table 3. SPRAINED baseline and updated prognostic models' equations.

Baseline SPRAINED prognostic model:

Y = -3.68 + (0.02 × age) + (0.02 × BMI) + (0.01 × pain when resting) + (0.01 × pain when bearing weight) + (0.61 if days from injury to assessment > 2) – (0.56 if able to bear any weight on the injured ankle) + (0.84 if the injury is a recurrent sprain)

Updated SPRAINED prognostic model (baseline + 4-weeks predictors):

Y = -4.4 + (0.01 × age) + (0.02 × BMI) + (0.01 × pain when resting) + (0.01 × pain when bearing weight) + (0.59 if days from injury to assessment > 2) – (0.68 if able to bear any weight on the injured ankle) + (0.99 if the injury is a recurrent sprain) + (0.02 × pain when bearing weight 4 weeks after injury)

The log odds (Y) estimated with these equations must be converted into a probability (P), which can be done by applying the following equation:

$$P = 1/(1 + \exp(-Y))$$

For peer review only

TRIPOD Checklist: Prediction Model Development and Validation

Section/Topic	Item		Checklist Item	Page
**Title and abstract**				
Title	1	D;V	Identify the study as developing and/or validating a multivariable prediction model, the target population, and the outcome to be predicted.	1
Abstract	2	D;V	Provide a summary of objectives, study design, setting, participants, sample size, predictors, outcome, statistical analysis, results, and conclusions.	2
**Introduction**				
Background and objectives	3a	D;V	Explain the medical context (including whether diagnostic or prognostic) and rationale for developing or validating the multivariable prediction model, including references to existing models.	4
	3b	D;V	Specify the objectives, including whether the study describes the development or validation of the model or both.	4
**Methods**				
Source of data	4a	D;V	Describe the study design or source of data (e.g., randomized trial, cohort, or registry data), separately for the development and validation data sets, if applicable.	4
	4b	D;V	Specify the key study dates, including start of accrual; end of accrual; and, if applicable, end of follow-up.	4-5
Participants	5a	D;V	Specify key elements of the study setting (e.g., primary care, secondary care, general population) including number and location of centres.	4-5
	5b	D;V	Describe eligibility criteria for participants.	4-5
	5c	D;V	Give details of treatments received, if relevant.	4
Outcome	6a	D;V	Clearly define the outcome that is predicted by the prediction model, including how and when assessed.	5
	6b	D;V	Report any actions to blind assessment of the outcome to be predicted.	5
Predictors	7a	D;V	Clearly define all predictors used in developing or validating the multivariable prediction model, including how and when they were measured.	5-7
	7b	D;V	Report any actions to blind assessment of predictors for the outcome and other predictors.	6
Sample size	8	D;V	Explain how the study size was arrived at.	6
Missing data	9	D;V	Describe how missing data were handled (e.g., complete-case analysis, single imputation, multiple imputation) with details of any imputation method.	6-7
Statistical analysis methods	10a	D	Describe how predictors were handled in the analyses.	6-7
	10b	D	Specify type of model, all model-building procedures (including any predictor selection), and method for internal validation.	6-7
	10c	V	For validation, describe how the predictions were calculated.	8
	10d	D;V	Specify all measures used to assess model performance and, if relevant, to compare multiple models.	7-8
	10e	V	Describe any model updating (e.g., recalibration) arising from the validation, if done.	7
Risk groups	11	D;V	Provide details on how risk groups were created, if done.	Not done
Development vs. validation	12	V	For validation, identify any differences from the development data in setting, eligibility criteria, outcome, and predictors.	4-5
**Results**				
Participants	13a	D;V	Describe the flow of participants through the study, including the number of participants with and without the outcome and, if applicable, a summary of the follow-up time. A diagram may be helpful.	8-9
	13b	D;V	Describe the characteristics of the participants (basic demographics, clinical features, available predictors), including the number of participants with missing data for predictors and outcome.	8-9
	13c	V	For validation, show a comparison with the development data of the distribution of important variables (demographics, predictors and outcome).	8-9
Model development	14a	D	Specify the number of participants and outcome events in each analysis.	7-8
	14b	D	If done, report the unadjusted association between each candidate predictor and outcome.	Not done
Model specification	15a	D	Present the full prediction model to allow predictions for individuals (i.e., all regression coefficients, and model intercept or baseline survival at a given time point).	Table 3
	15b	D	Explain how to the use the prediction model.	Suppl File 3
Model performance	16	D;V	Report performance measures (with CIs) for the prediction model.	9-10
Model-updating	17	V	If done, report the results from any model updating (i.e., model specification, model performance).	Not done
**Discussion**				
Limitations	18	D;V	Discuss any limitations of the study (such as nonrepresentative sample, few events per predictor, missing data).	11
Interpretation	19a	V	For validation, discuss the results with reference to performance in the development data, and any other validation data.	10
	19b	D;V	Give an overall interpretation of the results, considering objectives, limitations, results from similar studies, and other relevant evidence.	10-11
Implications	20	D;V	Discuss the potential clinical use of the model and implications for future research.	11-12
**Other information**				
Supplementary information	21	D;V	Provide information about the availability of supplementary resources, such as study protocol, Web calculator, and data sets.	12
Funding	22	D;V	Give the source of funding and the role of the funders for the present study.	12

TRIPOD Checklist: Prediction Model Development and Validation

*Items relevant only to the development of a prediction model are denoted by D, items relating solely to a validation of a prediction model are denoted by V, and items relating to both are denoted D;V. We recommend using the TRIPOD Checklist in conjunction with the TRIPOD Explanation and Elaboration document.

For peer review only

DEVELOPMENT AND PROSPECTIVE EXTERNAL VALIDATION OF A TOOL TO PREDICT POOR RECOVERY AT NINE MONTHS AFTER ACUTE ANKLE SPRAIN IN UK EMERGENCY DEPARTMENTS: THE SPRAINED PROGNOSTIC MODEL

Journal:	*BMJ Open*
Manuscript ID	bmjopen-2018-022802.R1
Article Type:	Research
Date Submitted by the Author:	05-Jun-2018

Complete List of Authors:	Schlussel, Michael; Centre for Statistics in Medicine, NDORMS<br>Keene, David; University of Oxford, Centre for Rehabilitation Research, Nuffield Departments of Orthopaedic Rheumatology and Musculoskeletal Sciences<br>Collins, Gary; University of Oxford, Centre for Statistics in Medicine<br>Bostock, Jennifer; Quality and Outcomes of Person-Centred Care Policy Research Unit<br>Byrne, Christopher; University of Plymouth, School of Health Professions, Faculty of Health and Human Sciences<br>Goodacre, Steve; University of Sheffield, Medical Care Research Unit<br>Gwilym, Stephen; University of Oxford, Oxford Trauma, Nuffield Departments of Orthopaedic Rheumatology and Musculoskeletal Sciences<br>Hagan, Daryl; University of Oxford, Centre for Rehabilitation Research, Nuffield Departments of Orthopaedic Rheumatology and Musculoskeletal Sciences<br>Haywood, Kirstie; University of Warwick, Warwick Research in Nursing, Division of Health Sciences, Warwick Medical School<br>Thompson, Jacqueline; University of Oxford, Centre for Rehabilitation Research, Nuffield Departments of Orthopaedic Rheumatology and Musculoskeletal Sciences<br>Williams, Mark; Oxford Brookes University, Sport and Health Sciences<br>Lamb, Sarah; University of Oxford, Ndorms

**Primary Subject Heading**:	Rehabilitation medicine
Secondary Subject Heading:	Emergency medicine, Evidence based practice
Keywords:	prognosis, clinical prediction rule, logistic model, ankle injuries, sprains and strains

SCHOLARONE™
Manuscripts

DEVELOPMENT AND PROSPECTIVE EXTERNAL VALIDATION OF A TOOL TO PREDICT POOR RECOVERY AT NINE MONTHS AFTER ACUTE ANKLE SPRAIN IN UK EMERGENCY DEPARTMENTS: THE SPRAINED PROGNOSTIC MODEL

Michael M Schlüssel [1*], David J Keene [2], Gary S Collins[1], Jennifer Bostock[3], Christopher Byrne[4], Steve Goodacre[5], Stephen Gwilym[6], Daryl A Hagan[2], Kirstie Haywood[7], Jacqueline Thompson[2], Mark A Williams[8], Sarah E Lamb [1,2] for the SPRAINED study team.

[1] Centre for Statistics in Medicine, Nuffield Departments of Orthopaedic Rheumatology and Musculoskeletal Sciences, University of Oxford, Oxford, United Kingdom

[2] Centre for Rehabilitation Research, Nuffield Departments of Orthopaedic Rheumatology and Musculoskeletal Sciences, University of Oxford, Oxford, United Kingdom

[3] Patient and Public Involvement representative, Quality and Outcomes of Person-Centred, Care Policy Research Unit, Canterbury, Kent, UK

[4] Faculty of Health and Human Sciences, University of Plymouth, Plymouth, United Kingdom

[5] School of Health and Related Research, University of Sheffield, Sheffield, United Kingdom

[6] Oxford Trauma, Nuffield Departments of Orthopaedic Rheumatology and Musculoskeletal Sciences, University of Oxford, Oxford, United Kingdom

[7] Warwick Research in Nursing, Division of Health Sciences, Warwick Medical School, University of Warwick, Coventry, United Kingdom

[8] Department of Sport, Health Sciences and Social Work, Oxford Brookes University, Oxford, United Kingdom

*** Corresponding author's address:**

Dr Michael Maia Schlüssel

Centre for Statistics in Medicine

Botnar Research Centre, Old Road

OX3 7LD

Oxford, United Kingdom

Email michael.schlussel@csm.ox.ac.uk

Telephone: +44 01865 737916

Word count: 4,222 words

For peer review only - http://bmjopen.bmj.com/site/about/guidelines.xhtml

ABSTRACT

OBJECTIVES: To develop and externally validate a prognostic model for poor recovery after ankle sprain. **SETTING AND PARTICIPANTS:** Model development used secondary data analysis from 584 participants in a UK multicentre randomised clinical trial. External validation used data from 682 participants recruited in 10 emergency departments across the UK for a prospective observational cohort. **OUTCOME AND ANALYSIS:** Poor recovery was defined as presence of pain, functional difficulty or lack of confidence in the ankle at 9-months after injury. Twenty-three baseline candidate predictors were included together in a multivariable logistic regression model to identify the best predictors of poor recovery. Relationships between continuous variables and the outcome were modelled using fractional polynomials. Regression parameters were combined over 50 imputed datasets using Rubin's rule. To minimise over-fitting, regression coefficients were multiplied by a heuristic shrinkage factor and the intercept re-estimated. Incremental value of candidate predictors assessed at 4-weeks after injury was explored using decision curve analysis and the baseline model updated. The final models included predictors selected based on the Akaike Information Criterion ($p<0.157$). Model performance was assessed by calibration and discrimination. **RESULTS:** Outcome rate was lower in the development (6.7%) than in the external validation dataset (19.9%). Mean age (29.9 and 33.6 years), BMI (26.3 and 27.1 kg/m^2), pain when resting (37.8 and 38.5 points) or bearing weight on the ankle (75.4 and 71.3 points) were similar in both datasets. Age, BMI, pain when resting, pain bearing weight, ability to bear weight, days until assessment, and injury recurrence were the selected predictors. The baseline model had fair discriminatory ability (c-statistic 0.72; 95%CI: 0.66-0.79) but poor calibration. The updated model presented better discrimination (c-statistic 0.78; 95%CI: 0.72-0.84), but equivalent calibration. **CONCLUSIONS:** The models include predictors easy to assess clinically and show benefit when compared to not using any model. **Registry number:** ISRCTN12726986.

Keywords: prognosis, clinical prediction rule, logistic model, ankle injuries, sprains and strains

For peer review only - http://bmjopen.bmj.com/site/about/guidelines.xhtml

STRENGTHS AND LIMITATIONS OF THIS STUDY

- This is the first study to develop and externally validate a tool to predict poor recovery after ankle sprain, including a wide range of clinically relevant candidate predictors.
- Despite containing information on the outcomes of interest and numerous prognostic variables, the development dataset was not originally acquired to build a prognostic model.
- The number of events in the development dataset was relatively small compared to the number of candidate predictors examined.
- Yet, the prognostic models were developed using robust statistical methods, adjusted for overfitting and reported according to the most recent relevant guidelines available.
- Generalisability of findings is enhanced by the multi-centre characteristic of the datasets used in the development and external validation of the models.

For peer review only

For peer review only - http://bmjopen.bmj.com/site/about/guidelines.xhtml

INTRODUCTION

Ankle sprains are one of the most common musculoskeletal injuries, representing up to 5% of all emergency department (ED) attendances in the UK.**[1]** Despite heterogeneity in sampling frame (e.g. restricted to elite athletes or excluding older people), inception, and follow-up time points, studies have indicated that approximately 30% of people have persistent problems one year after ankle sprain.**[2, 3]** In a large multi-centre randomised clinical trial conducted in the UK, a similar proportion (30%) of participants had poor outcome at 9 months.**[4]** Other studies indicate a recovery plateau at around 9 months, and residual disability after this point to be persistent.**[5]**

In the acute phase after a sprain, physical examination of the ankle is often difficult due to swelling and pain. Predicting prognosis at this stage is uncertain and based on clinical judgement. When concerned about the injury severity, clinicians operate a system of review within one week in a trauma clinic (or equivalent service), which allows some resolution of swelling and reassurance about the presence of other significant mechanical derangement.**[6]** The Ottawa ankle rule is also an alternative to reduce the requirement for imaging without missing important fractures.**[7]**

In 2008, Van Rijn et al conducted a systematic review on the clinical pathway and prognostic factors of ankle sprain recovery and found a single eligible study concluding that high levels of sports activity have prognostic value for residual symptoms.[2] In a more recent systematic review, we have identified nine studies reporting results for baseline prognostic factors of recovery after an acute ankle sprain.[8] Age, gender, swelling, range of motion, weight bearing ability, pain, injury severity, palpation/stress score, injury mechanism, self-reported recovery, re-sprain, MRI determined number of sprained ligaments and bone bruise were reported as independent predictors of poor recovery. However, almost all studies performed poorly on the risk of bias assessment, mainly due to incomplete or inadequate reporting standards for study participants, attrition, methods of assessment for predictors, confounding and statistical methods used, so results should be interpreted with caution.

To the best of our knowledge, there are no externally validated prognostic models for recovery after acute ankle sprain. Polzer et al. developed an algorithm to help clinicians with

For peer review only - http://bmjopen.bmj.com/site/about/guidelines.xhtml

the diagnosis and treatment of acute ankle injuries, but this is considerably based on expert judgements and do not use currently recommended methods for the development of prognostic models.[9] A robustly developed and validated prognostic model could help to target treatment better and improve outcomes for people who have an ankle sprain.[10] Therefore, the development of a new prognostic model, considering a range of plausible candidate predictors, and ideally with the evaluation of its performance on an external dataset (external validation), is indicated.

The aim of our study was to develop and externally validate the SPRAINED (Synthesising a clinical Prognostic Rule for Ankle Injuries in the Emergency Department) prognostic model, to identify people at risk of poor recovery at nine months after acute ankle sprain.

METHODS

Study populations and data collection

Data from the Collaborative Ankle Support Trial (CAST), were used to develop the prognostic model.**[11]** CAST was a pragmatic multicentre randomised controlled trial on the effectiveness of different mechanical ankle supports compared with a double-layer tubular compression bandage for managing severe ankle sprains. The trial sample comprised 584 participants aged 16 years or older, with an ankle sprain of grade 2 or 3, attending eight EDs in the UK between April 2003 and July 2005, within 7 days after their injury, and were not able to fully bear weight on the injured ankle at baseline. Further data was collected at 4 and 12 weeks, and 9 months after randomisation. The CAST methods and a CONSORT flow diagram are available elsewhere.**[11]**

To assess the model's performance in an external population, the SPRAINED prospective observational cohort was recruited. Participants were aged 16 years or above, with acute ankle sprains of any grade, attending 10 NHS EDs across England, within 7 days after their injury. Patients were excluded if they presented with an ankle fracture (except flake fractures < 2mm) or any other recent (< 3 months) lower limb fracture. Participants were not randomised, nor did they receive any interventions other than usual care at each site. The study recruited 682 participants between July 2015 and March 2016. Data collection covered clinical and socio-demographic information collected at ED presentation (baseline), with follow-up assessments at 4 weeks, 4 and 9 months after the initial injury, either by self-reported paper-based forms sent back to the study office by postal mail, electronic

For peer review only - http://bmjopen.bmj.com/site/about/guidelines.xhtml

questionnaires, or telephone interviews. The SPRAINED questionnaires included all variables selected as predictors in the model and the components of the outcome of interest. All participants of both studies have provided written informed consent before any data collection took place. Ethics approval was from the National Research Ethics Committee (REC) (London - Chelsea), REC number 15/LO/0538, on 10th April 2015. The study protocol was registered on 30th April 2015; registry number ISRCTN12726986.

Definition of outcome

A prognostic model was developed to predict 'poor recovery' at 9 months after an acute ankle sprain. Poor recovery was defined as the presence of pain, lack of confidence in the ankle (persistent feeling of giving way) or functional difficulty.**[12, 13]** The presence of these symptoms was assessed by patient-reported responses given to specific items (P1, Q3 and Q4) of the Foot and Ankle Outcome Score (FAOS).**[14]** Participants who answered one or more of these questions with any of the two most extreme response options ("daily" or "always" P1; "severely" or "extremely" for Q3 or Q4) were considered to have poor outcome.

Baseline candidate predictors

Thirty-two baseline candidate predictors were considered plausible predictors of poor outcome and pre-selected from a pool of 170 variables available in the CAST dataset (**Supplemental Tables 1 and 2**). This initial selection was made internally by the research team, taking into account the results from our systematic literature review **[8]** and the conclusions from a consensus group meeting convened for the SPRAINED study, which included clinicians, medical researchers, statisticians and PPI representatives. The 32 candidate predictors included socio-demographic information (e.g. age, sex, body mass index (BMI), education, employment status); pre-injury quality of life, mobility and lifestyle indicators (e.g. engagement in sports activities); clinical data on injury presentation; baseline (post-injury) mobility levels, pain and weight-bearing status (**Supplemental Table 3).**

At this stage, variables were excluded or combined before statistical modelling if they had 60% or more of missing information; displayed high collinearity ($r \geq 0.8$) with another candidate predictor; presented empty or low cell counts ($n < 5$) when tabulated against the

For peer review only - http://bmjopen.bmj.com/site/about/guidelines.xhtml

outcome; were the offending variable causing perfect prediction during the multiple imputation process (**Supplemental Table 4; Supplemental Figure 1**).

Sample size considerations

It is widely recommended that the dataset used to develop a prognostic tool should contain a minimum of 5-10 outcome events per variable (EPV) included as a predictor in the model.**[15-20]** After the exclusion of nine baseline candidate predictors for the reasons described above, 23 variables from baseline remained as candidate predictors. However, some of these predictors were categorical variables with more than two levels, so we ended with 35 candidate parameters, meaning the EPV ratio was approximately three.

As to the best of our knowledge this was the first study aiming to develop prediction models to assess the risk of poor recovery after an acute ankle sprain, we opted for relaxing the EPV rule in favour of including more potentially important predictors. Nevertheless, we adopted several strategies to minimise bias and overfitting, as described below.

Descriptive analysis

Baseline and 4-week follow-up characteristics of the CAST and SPRAINED participants were summarised using means, standard deviations (SDs) and ranges for continuous variables, or counts and percentages for categorical variables. Inspection of extreme values (outliers) took place to confirm whether they were clinically plausible and visual assessment of data distribution for continuous predictors in both datasets was conducted. No formal statistical tests were performed to compare the values between the studies.

Prognostic model development

Using logistic regression, we developed the prognostic model to predict the probability of poor recovery. We performed multiple imputation using chained equations (MICE) **[21]** to handle missing data, with 50 imputed datasets created. Continuous variables were kept as continuous to avoid loss of prognostic information,**[22]** and the shape of their relationship with the outcome studied and modelled with nonlinear functions such as fractional polynomials (FPs) where appropriate.**[23]** As several continuous variables were included in the models, we used the multivariable fractional polynomial (MFP) algorithm.**[24, 25]**

For peer review only - http://bmjopen.bmj.com/site/about/guidelines.xhtml

Multiple imputation and fractional polynomials were combined using the *mfpmi* function in Stata.**[26]** The estimated regression parameters (coefficients and variances) were combined over the 50 imputed datasets using Rubin's rule.**[27, 28]** After identifying the best transformation terms for continuous variables, the final model included predictors (and respective transformations, where applicable) selected from the full multivariable model with all candidate predictors based on the Akaike Information Criterion (equivalent to a p-value < 0.157).**[29]** To adjust for over-fitting, due to small EPV, we multiplied all regression coefficients by the heuristic shrinkage factor,**[30]** then re-estimated the intercept. All model assumptions were checked and differences between incomplete and imputed datasets inspected. Imputed data from all 584 participants were included in all analyses.

Incremental value analysis and model update

In addition to the baseline predictors, 14 additional variables from the CAST 4-weeks follow-up questionnaire were also selected as potential predictors that could increase the model's prognostic ability (**Supplemental Table 3**). First, all additional 4-weeks candidate predictors were included together in the final baseline model and only those achieving a p-value < 0.157 were considered for inclusion in the updated model (i.e. a model including baseline and 4-weeks predictors). Finally, the updated model was compared with the original baseline model using decision curve analysis (DCA) plots to determine whether the inclusion of additional predictors reflected in increased net benefit.**[31, 32]**

External validation: Model performance

We assessed the model performance in the prospectively collected SPRAINED cohort. Missing data in the SPRAINED cohort was handled using MICE, creating 50 imputed datasets. Performance was evaluated by assessing calibration and discrimination. Calibration is the agreement between observed and predicted probabilities of poor outcome. Calibration was assessed graphically using calibration plots, with observed risks plotted on the *y*-axis against predicted risks on the *x*-axis.**[33, 34]** The calibration plot was created by regressing the outcome on the predicted probability using a locally weighted scatter plot smoother (lowess). The calibration plot was also supplemented with estimates of the calibration slope and intercept. Models with perfect calibration will have a calibration slope of 1 and intercept 0 (i.e., prediction lying on the 45° line). Calibration plots followed

For peer review only - http://bmjopen.bmj.com/site/about/guidelines.xhtml

recommendations of overlaying calibration curves from each imputed data set.**[35]** Discrimination reflects the ability of the model to distinguish between participants who did and did not experience an event during the study period. Discrimination was assessed using the c-statistic, where a value of 0.5 represents chance and 1 represents perfect discrimination.**[36]** Finally, to estimate the benefit of using the developed models, patients were ranked according to their estimated risks. These were used to calculate the number of people per 1000 identified as being at high risk according to selected thresholds and how many of these went on to present the outcomes compared with not using the model. Individual probabilities of developing the outcomes were estimated by applying the developed prognostic models to each participant in the SPRAINED imputed datasets. We assessed the performance of both the baseline and updated models using imputed data from all 682 participants.

Patient involvement

A PPI representative was involved in the study from the beginning, providing advice on key aspects of the study design, including the definition of the research question, choice of the outcome and selection of relevant candidate predictors during the consensus group meeting. They will be consulted for the public dissemination of any product arriving from this research.

Reporting

We followed the TRIPOD Statement for the reporting of our study.**[37]**

RESULTS

Baseline characteristics for the CAST (development) and SPRAINED (validation) cohorts are summarised in **Table 1**. On average, participants were slightly older in SPRAINED than in CAST. Participants in SPRAINED had an average BMI within the overweight category, likewise those in CAST. The mean pain scores when resting or bearing weight on the ankle of SPRAINED participants were also similar to those observed for CAST participants. Differently from CAST, in SPRAINED about half of participants were female, the majority presented to an ED within 2 days from injury for assessment and were able to bear some weight on their injured ankles (**Table 1**).

For peer review only - http://bmjopen.bmj.com/site/about/guidelines.xhtml

Table 1. Baseline characteristics of the participants in the CAST trial and SPRAINED prospective observational cohort.

Variable	CAST Trial		SPRAINED Cohort	
	**Mean (SD)**	**Min - Max**	**Mean (SD)**	**Min - Max**
Age (years)	29.88 (10.77)	16 – 72	33.62 (13.38)	16 – 89
Height (m)	1.73 (0.98)	1.47 – 2.01	1.72 (1.02)	1.50 – 2.01
Weight (kg)	78.56 (15.44)	39.92 – 133.36	80.44 (18.13)	44.50 – 180
Body mass index (kg/m^2)	26.34 (5.19)	16.07 – 53.77	27.08 (5.70)	17.31 – 64.30
Pain when resting (score)	37.75 (23.49)	0 – 100	38.50 (22.50)	0 – 100
Pain when bearing weight (score)	75.42 (19.61)	0 – 100	71.30 (21.00)	0 – 100
	**Frequency**	**%**	**Frequency**	**%**
Sex				
Male	337	57.71	327	47.95
Female	247	42.29	355	52.05
Days from injury to assessment				
0-2	118	44.87	614	90.03
3 or more	145	55.13	68	9.97
Able to bear weight at Baseline assessment				
No	446	77.03	179	26.44
Yes	133	22.97	498	73.56
Recurrent sprain				
No	517	90.38	583	91.38
Yes	55	9.62	55	8.62
Current employment				
None	132	22.60	161	23.68
Part time	92	15.75	92	13.53
Full time	360	61.64	427	62.79
Injury mechanism				
At home	99	18.00	144	21.56
Practicing sports	203	36.91	230	34.43
At work	79	14.36	91	13.62
Outside, in public	169	30.73	203	30.39

Table 2 shows the rates of poor recovery in the CAST trial and SPRAINED cohort datasets, as well as the number of its component symptoms, at 9 months after injury. There was a lower rate of poor recovery in the SPRAINED cohort than observed in the CAST trial, but the percentage of missing data for the outcome was similar in both studies.

For peer review only

For peer review only - http://bmjopen.bmj.com/site/about/guidelines.xhtml

Table 2. Outcome and respective symptoms components rates and proportion of missing data in the CAST trial and SPRAINED prospective observational cohort.

	Pain	Lack of confidence	Instability	Poor recovery	Missing data	TOTAL[1]
CAST	84 (14.4%)	42 (7.2%)	67 (11.5%)	116 (19.9%)	144 (24.7%)	584
SPRAINED	3 (0.4%)	23 (3.4%)	37 (5.4%)	46 (6.7%)	155 (22.7%)	682

Note: Poor recovery defined as the presence of one or more of the following symptoms: pain, lack of confidence or instability/difficulty with the ankle.

Table 3 displays the summary of the final multivariable models (predictor's coefficients, respective 95% confidence intervals and p-values). Seven of the 23 baseline candidate predictors were selected for inclusion in the baseline model: age, BMI, pain when resting, pain when bearing weight, days from injury to assessment, ability to bear weight and whether or not the injury was a recurrent sprain. The best fit for all continuous predictors were linear transformations (mean subtractions) and were later incorporated into the model by updating the intercept accordingly (**Supplemental Table 5**).

For peer review only

For peer review only - http://bmjopen.bmj.com/site/about/guidelines.xhtml

Table 3. Summary of the final baseline and updated (baseline plus 4-weeks predictors) logistic regression models and respective shrunk coefficients and intercepts.

Predictors	Baseline model					Updated model (baseline plus 4-weeks predictors)				
	Coefficient	95% CI		p	Shrunk coefficient	Coefficient	95% CI		P	Shrunk coefficient
Age	0.027	0.006	0.048	0.014	0.019	0.018	-0.005	0.040	0.127	0.015
BMI	0.031	-0.014	0.076	0.178	0.022	0.025	-0.022	0.072	0.292	0.021
Pain when resting	0.016	0.005	0.027	0.005	0.011	0.010	-0.002	0.022	0.107	0.008
Pain when bearing weight	0.019	0.004	0.035	0.016	0.014	0.014	-0.002	0.030	0.092	0.012
Pain when bearing weight 4 wks. after injury	-	-	-	-	-	0.022	0.012	0.032	< 0.001	0.018
Days from injury to assessment (reference 0-2)										
3 or more	0.854	0.068	1.640	0.034	0.605	0.702	-0.117	1.520	0.092	0.591
Able to bear weight at Baseline (reference No)										
Yes	-0.792	-1.376	-0.207	0.008	-0.561	-0.802	-1.412	-0.192	0.010	-0.676
Recurrent sprain (reference No)										
Yes	1.180	0.417	1.944	0.003	0.836	1.170	0.386	1.953	0.004	0.985
Intercept	-1.580	-2.152	-1.008	< 0.001	-1.363	-1.543	-2.128	-0.958	< 0.001	-1. 420

95% CI: 95% confidence interval

Linear terms selected by the MFP for continuous predictors: Age – 29.88; BMI – 26.32; Pain when resting – 37.75; Pain when bearing weight – 75.40; Pain when bearing weight at 4 weeks after injury – 36.23.

For peer review only - http://bmjopen.bmj.com/site/about/guidelines.xhtml

Only pain when bearing weight on the sprained ankle at 4 weeks after injury was included in the updated model (baseline plus 4-week predictors) (**Table 3**). By inspecting the DCA plots shown in **Figure 1** it is possible to see a clear net benefit gain over the entire range of thresholds when using the updated prognostic model in comparison to the baseline model or considering all patients (or no patient) at risk of having poor recovery after an acute ankle sprain.

Shrinkage suggested both prognostic models (baseline and updated) had predictor-outcome associations that were too large. The heuristic shrinkage factor for the coefficients of the predictors in the baseline prognostic model was 0.71. For the updated version (baseline plus 4-weeks predictors), the estimated heuristic shrinkage factor was 0.84. The shrunk coefficients and intercepts for the final models are presented in **Table 3**.

Overall, discrimination of the baseline model was fair, with a c-statistic of 0.72 (95%CI: 0.66 to 0.79). Calibration of the baseline prognostic model in the external validation dataset was poor though, as can be evidenced by inspecting the calibration plot with overlaid calibration lines from the 50 imputed datasets (**Figure 2**). The calibration slope was 1.13 (95%CI: 0.76 to 1.5) and the calibration intercept was -0.71 (95%CI: -0.98 to -0.44). The updated model (baseline plus 4-weeks predictors) presented better discriminatory ability in the SPRAINED dataset than the baseline model (c-statistic = 0.78; 95%CI: 0.72 to 0.84), but equivalent calibration, with an intercept closer to zero (-0.51; 95%CI: -0.78 to -0.24) and slope slightly further from one (1.17; 95%CI: 0.86 to 1.48).

Table 4 shows how many of 1000 people would be identified as being at high risk (based on thresholds of 5, 10, 15, and 20%) using the developed prognostic models, and how many of these would actually present poor recovery 9 months after an acute ankle sprain. There seems to be little difference between the baseline and updated models, with both identifying similar numbers of patients who would experience a poor outcome after an acute ankle sprain. However, less patients are deemed at high risk by using the updated model for (less false positives) across all thresholds of predicted probability suggesting that reassessing the patients at 4 weeks after the injury might be beneficial to a more accurate prediction of their probability of poor outcome. Using any of the models is clearly beneficial, when compared to not using any model (i.e. considering all patients – or no patients – as high risk of developing poor outcome).

For peer review only - http://bmjopen.bmj.com/site/about/guidelines.xhtml

Table 4. Models performance (numbers at risk and outcomes identified) at varying risk thresholds for 1000 patients.

Selected thresholds	Number of patients at risk		Number of events	
	High risk	Low risk	Identified	Not identified
Consider all high risk	1000	0	85	0
Predicted probability as per baseline model				
≥5%	971	39	85	0
≥10%	797	203	74	11
≥15%	543	457	63	22
≥20%	351	649	52	33
Predicted probability as per updated model (baseline plus 4-weeks predictors)				
≥5%	882	118	85	0
≥10%	517	483	71	14
≥15%	358	642	56	29
≥20%	259	741	41	44

DISCUSSION

We developed a prognostic model to predict a composite outcome representing the presence of at least one of the following symptoms at 9 months after an acute ankle sprain: pain, functional difficulty or lack of confidence in the ankle. The model presented fair discriminatory ability in a prospectively collected external validation cohort, but poor calibration. Including an additional variable collected at 4 weeks after the injury (pain when bearing weight on the injured ankle) improved the discriminatory ability and calibration of the model. The models included predictors that are easily collected and provided reasonable predictions of poor recovery for patients with acute ankle sprain.

In a recent systematic review, we have reported that some of the variables selected for inclusion in our prognostic model, have been previously identified as important predictors of short, medium or long term recovery after ankle sprain.[8] According to O'Connor et al. age and weight bearing ability are predictors of ankle function, as measured by the Karlsson function score, both at 4 weeks and 4 months after injury.[38] Akacha et al. also demonstrated that age was an important predictor of slower and incomplete recovery after ankle sprain, as measure by the Foot and Ankle Outcome Score.[39] The magnitude of pain at rest at 3 months has also been shown to have prognostic value for poorer self-reported recovery at 12 months after ankle sprain by Van Middelkoop et al.[40] On the other hand, Findings regarding recurrence of ankle sprain are conflicting. McKeon et al., reported that recurrent ankle sprain was not a significant predictor of time to return-to-play after an ankle injury.[41] This is contrary to reports of an association between recurrent sprains and

For peer review only

chronic ankle instability reported in a systematic review conducted by Pourkazemi et al. [42] One possible explanation for these contradictory results may be the nature of the outcomes investigated in each study. When more subjective aspects of recovery (such as ankle function or instability) are considered in the definition of the endpoint, like in the present study, re-spraining the ankle seems to be an important predictor of recovery.

The inclusion of BMI in the prognostic model is another issue that deserves consideration. Although not statistically significant in the final multivariable logistic regression analysis, according to AIC (p<0.157), we have decided to keep BMI in the model for several reasons. First, this decision prevented another round of predictor selection, which could increase over-fitting. The model building process was not solely based on statistical rationale, and BMI was considered to be an important predictor by clinicians during our consensus group meeting. BMI is an easy to assess surrogate measure of body weight that is frequently collected at clinical routine and one that most patients know how to calculate themselves. Finally, its inclusion does not add much complexity to the models.

To the best of our knowledge, this is the first study to develop and externally validate a prognostic model to predict a clinically relevant outcome in people with acute ankle sprains exploring a wide range of clinically plausible candidate predictors. We used robust statistical methods to select the predictors and assess the model´s performance in a large external prospective cohort. Generalisability of the findings are enhanced by the multi-centre data from the CAST and SPRAINED cohorts that represented a range of district general and major trauma centres. The observational cohort we prospectively recruited for SPRAINED is representative of patients presenting to EDs in the UK. We followed the most recent and complete guidelines available on the reporting of prognostic model development,**[37]** and applied recommended methods to minimise overfitting. For example, continuous variables, whenever possible, were kept as continuous to avoid loss of information. Nonlinear relationships were investigated using the best variables transformations found by multivariable fractional polynomials. The study included an internal correction for model optimism (shrinkage of regression coefficients and re-estimation of intercepts) as well as a prospective external validation phase. The amount of missing data in the external validation dataset, which is commonplace in studies of this nature, was considerably smaller than that

For peer review only - http://bmjopen.bmj.com/site/about/guidelines.xhtml

observed in the development dataset. Finally, we performed missing data imputation to produce a set of 50 complete datasets and enable robust analyses.

Limitations of the SPRAINED study are acknowledged. Firstly, data used to develop the prognostic models were from a prior randomised controlled trial (CAST), so were not originally intended to fulfil this aim. However, the CAST cohort did represent the best dataset available, with information on the symptoms and clinical events of interest, and a wide range of the candidate prognostic variables considered to have predictive ability. Secondly, the CAST dataset used to develop the prognostic model was relatively small compared to the number of candidate predictors.**[15-20]** As previously highlighted, the low EPV observed for the two developed models might have contributed to the optimism found for both and, therefore, to the poor calibration on the external validation dataset. Thirdly, the amount of missing data observed in the development dataset. Because of that, a number of candidate predictors were omitted before the process of data imputation, to avoid instability of the imputation models. Therefore, some important predictors could have conceivably been missed in the development phase of the SPRAINED study. Finally, the rates of poor outcome in the SPRAINED cohort were lower than in the CAST trial and those reported in previous systematic reviews.**[2, 3]** These variations in poor outcome rates and clinically important differences in baseline characteristics included in the prognostic model (such as days from injury to clinical assessment and ability to bear weight on the injured ankle) highlight the issue of different sampling frames.

Clinical examination of acute ankle sprain is challenging as tolerance of physical examination tests is often poor due to pain and swelling. Imaging is often not routinely available. A prognostic tool could enable better targeting of treatments such as immobilisation casts, which although effective can be inconvenient to patients, to those deemed at low risk of poor outcome. On the other hand, it has the potential to help clinicians targeting treatments such as surgery and physiotherapy to patients who are at highest risk of poor outcome.

The SPRAINED prognostic model benefits from including predictors that are easy to measure, and usually assessed in clinical routine. Given the herby discussed limitations in its predictive performance, we suggest that its value would be in assisting the clinician to estimate the probability of a poor outcome, instead of being used as a decision making tool

For peer review only - http://bmjopen.bmj.com/site/about/guidelines.xhtml

in isolation. Improved predictive performance of the models with the addition of information on pain when bearing weight at 4 weeks indicates that re-assessment of prognosis after the acute phase is worth consideration for patients initially deemed to have elevated probability of delayed recovery. Besides, as it is an easy-to-use instrument, patients themselves can estimate their probability of poor outcome and gain some reassurance in their decisions to seek for further medical assistance or not.

If implemented in clinical practice, clinicians should be aware that there is a degree of uncertainty associated to the calculated risk of poor outcome when using the SPRAINED prognostic model. This uncertainty can lead to over or under referral of patients to review clinics or referral treatment such as physiotherapy. Future work could examine how well the model performs in comparison (or addition) to the clinician impression. Moreover, we recommend further research to evaluate the impact of using the SPRAINED prognostic model in clinical practice to predict patient outcomes and to assess the acceptability and uptake of the tool by clinicians in the EDs.

In conclusion, the SPRAINED prognostic models performed reasonably and despite some miscalibration show benefit in identifying patients at high risk of poor outcome after an acute ankle sprain. The models may assist clinical-decision making when assessing and advising people with ankle sprains in the ED setting and when deciding on on-going management. The models benefit from using predictors that are simple to obtain during routine clinical assessment.

DATA SHARING

All data requests should be submitted to the corresponding author for consideration. Access to anonymised data may be granted following review. Exclusive use will be retained until the publication of major outputs.

FUNDING

The SPRAINED study was funded by the National Institute of Health Research (NIHR) Health Technology Assessment programme (project number 13/19/06). Supported by the NIHR Biomedical Research Centre, Oxford, and the NIHR Fellowship programme (Dr David Keene, PDF-2016-09-056). Sarah Lamb receives funding from the National Institute for Health Research (NIHR) Collaboration for Leadership in Applied Health Research and Care Oxford at

For peer review only - http://bmjopen.bmj.com/site/about/guidelines.xhtml

403 Oxford Health NHS Foundation Trust. The views and opinions expressed therein are those of 404 the authors and do not necessarily reflect those of the Health Technology Assessment 405 programme, NIHR, NHS or the Department of Health.

406 **COMPETING INTERESTS**

407 None declared.

For peer review only

AUTHOR'S CONTRIBUTIONS

MMS analysed and interpreted the data, and led the writing of the manuscript. DJK had substantial contribution in data acquisition, analysis and interpretation. GSC had substantial contribution in the study conception and design, data analysis and interpretation. JB had substantial contribution in the study conception and design. CB had substantial contribution in the data acquisition. SG had substantial contribution in the study conception and design. SG had substantial contribution in the study conception and design. DAH had substantial contribution in the data acquisition. KH had substantial contribution in the study conception and design. JT had substantial contribution in the data acquisition. MAW had substantial contribution in the study conception and design, data acquisition. SEL was responsible for the study conception and design, had substantial contribution in data interpretation. All authors revised and approved the final version of the manuscript.

ACKNOWLEDGEMENTS

The SPRAINED study team members are: Sarah E Lamb (Chief Investigator); David J Keene (Study Lead); Gary S Collins, Mark Williams, Steve Goodacre, Matthew Cooke, Stephen Gwilym, Phil Hormbrey, David Wilson, Jennifer Bostock (Co-investigators); Daryl Hagan (Study administrator); Damian Haywood (Senior study manager); Jacqueline Thompson, Christopher Byrne (Research Physiotherapists); Michael Maia Schlüssel (Study statistician); Philip Hormbrey, Susan Dorrian, Steve Goodacre, Victoria Stacey, Tim Coats, Sarah Wilson, Jason Kendall, David Clarke, Antoanela Colda, Deborah Mayne (Principal Investigators and their clinical and research teams at collaborating recruitment centres); Kirstie Haywood (consultation and senior facilitation of the consensus meeting). We would like to thanks Prof. Richard Riley, Prof. Kevin Mackway-Jones and Prof. Suzanne McDonough from the Independent Study Steering Committee for their invaluable comments, support and advise throughout the study. We also acknowledge the contributions of Vicki Barber and the staff members at Oxford Clinical Trials Research Unit and Centre for Rehabilitation Research for their support in delivering the SPRAINED study; the members of the consensus group meeting, including the PPI representatives; and all those who have in some way contributed to the conception, conduction and reporting of the SPRAINED study.

For peer review only

REFERENCES

1. Wilson RW, Gansneder BM. Measures of functional limitation as predictors of disablement in athletes with acute ankle sprains. *J Orthop Sports Phys Ther* 2000;30:528-35.

2. van Rijn RM, van Os AG, Bernsen RM, et al. What is the clinical course of acute ankle sprains? A systematic literature review. *Am J Med* 2008;121:324-31.

3. Polzer H, Kanz KG, Prall WC, et al. Diagnosis and treatment of acute ankle injuries: development of an evidence-based algorithm. *Orthop Rev* (Pavia) 2012;4:e5.

4. Cooke MW, Marsh JL, Clark M, et al. Treatment of severe ankle sprain: a pragmatic randomised controlled trial comparing the clinical effectiveness and cost-effectiveness of three types of mechanical ankle support with tubular bandage. The CAST trial. Health technology assessment 2009;13:iii, ix-x, 1-121.

5. Verhagen RA, de Keizer G, van Dijk CN. Long-term follow-up of inversion trauma of the ankle. *Arch Orthop Trauma Surg* 1995;114:92-6.

6. van Dijk CN, Mol BW, Lim LS, Marti RK, Bossuyt PM. Diagnosis of ligament rupture of the ankle joint. Physical examination, arthrography, stress radiography and sonography compared in 160 patients after inversion trauma. Acta orthopaedica Scandinavica 1996;67:566-70.

7. Stiell I, Wells G, Laupacis A, Brison R, Verbeek R, Vandemheen K, et al. Multicentre trial to introduce the Ottawa ankle rules for use of radiography in acute ankle injuries. Multicentre Ankle Rule Study Group. Bmj 1995;311:594-7.

8. Thompson JY, Byrne C, Williams MA, et al. Prognostic factors for recovery following acute lateral ankle ligament sprain: a systematic review. *BMC Musculoskelet Disord*. 2017;18:421.

9. Polzer H, Kanz KG, Prall WC, Haasters F, Ockert B, Mutschler W, et al. Diagnosis and treatment of acute ankle injuries: development of an evidence-based algorithm. Orthopedic reviews 2012;4:e5.

For peer review only - http://bmjopen.bmj.com/site/about/guidelines.xhtml

10. Hemingway H, Croft P, Perel P, Hayden JA, Abrams K, Timmis A, et al. Prognosis research strategy (PROGRESS) 1: A framework for researching clinical outcomes. Bmj 2013;346:e5595.

11. Lamb SE, Marsh JL, Hutton JL, et al. Mechanical supports for acute, severe ankle sprain: a pragmatic, multicentre, randomised controlled trial. *Lancet* 2009;373:575-81.

12. van Rijn RM, Willemsen SP, Verhagen AP, et al. Explanatory variables for adult patients' self-reported recovery after acute lateral ankle sprain. *Phys Ther* 2011;91:77-84.

13. Wikstrom EA, Hubbard-Turner T, McKeon PO. Understanding and treating lateral ankle sprains and their consequences: a constraints-based approach. *Sports Med* 2013;43:385-93.

14. Roos EM, Brandsson S, Karlsson J. Validation of the foot and ankle outcome score for ankle ligament reconstruction. *Foot Ankle Int* 2001;22:788-94.

15. Harrell Jr FE, Lee KL, Califf RM, et al. Regression modelling strategies for improved prognostic prediction. *Stat Med* 1984;3:143-52.

16. Harrell Jr FE, Lee KL, Mark DB. Multivariable prognostic models: issues in developing models, evaluating assumptions and adequacy, and measuring and reducing errors. *Stat Med* 1996;15:361-87.

17. Peduzzi P, Concato J, Kemper E, et al. A simulation study of the number of events per variable in logistic regression analysis. *J Clin Epidemiol* 1996;49:1373-9.

18. Moons KG, de Groot JA, Bouwmeester W, et al. Critical Appraisal and Data Extraction for Systematic Reviews of Prediction Modelling Studies: The CHARMS Checklist. *PLoS med* 2014;11:e1001744.

19. Pavlou M, Ambler G, Seaman SR, et al. How to develop a more accurate risk prediction model when there are few events. *BMJ* 2015;351:h3868.

20. Vittinghoff E, McCulloch CE. Relaxing the rule of ten events per variable in logistic and Cox regression. *Am J Epidemiol* 2007;165:710-8.

21. White IR, Royston P, Wood AM. Multiple imputation using chained equations: Issues and guidance for practice. *Stat Med* 2011;30:377-99.

For peer review only

For peer review only - http://bmjopen.bmj.com/site/about/guidelines.xhtml

22. Collins GS, Ogundimu EO, Cook JA, et al. Quantifying the impact of different approaches for handling continuous predictors on the performance of a prognostic model. *Stat Med* 2016;35:4124-35.

23. Royston P, Altman DG. Regression Using Fractional Polynomials of Continuous Covariates: Parsimonious Parametric Modelling. *J R Stat Soc Ser C Appl Stat* 1994;43:429-67.

24. Royston P, Sauerbrei W. MFP: Multivariable Model-Building with Fractional Polynomials. In: Multivariable Model-Building: John Wiley & Sons, Ltd; 2008:115-50.

25. Sauerbrei W, Royston P, Binder H. Selection of important variables and determination of functional form for continuous predictors in multivariable model building. *Stat Med* 2007;26:5512-28.

26. Morris TP, White IR, Carpenter JR, et al. Combining fractional polynomial model building with multiple imputation. *Stat Med* 2015;34:3298-317.

27. Marshall A, Altman DG, Holder RL, et al. Combining estimates of interest in prognostic modelling studies after multiple imputation: current practice and guidelines. *BMC Med Res Methodol* 2009;9:57.

28. Rubin DB. Multiple Imputation for Nonresponse in Surveys: Wiley; 2004.

29. Atkinson AC. A note on the generalized information criterion for choice of a model. *Biometrika* 1980;67:413-8.

30. van Houwelingen JC, LeCessie S. Predictive value of statistical models. *Stat Med* 1990;9:1303-25.

31. Vickers AJ, Elkin EB. Decision curve analysis: a novel method for evaluating prediction models. *Med Decis Making* 2006;26:565-74.

32. Vickers AJ, Van Calster B, Steyerberg EW. Net benefit approaches to the evaluation of prediction models, molecular markers, and diagnostic tests. *BMJ* 2016;352:i6.

33. Austin PC, Steyerberg EW. Graphical assessment of internal and external calibration of logistic regression models by using loess smoothers. *Stat Med* 2014;33:517-35.

For peer review only - http://bmjopen.bmj.com/site/about/guidelines.xhtml

34. Wood AM, Royston P, White IR. The estimation and use of predictions for the assessment of model performance using large samples with multiply imputed data. *Biom J* 2015; 10.1002/bimj.201400004.

35. Janssen KJ, Moons KG, Kalkman CJ, et al. Updating methods improved the performance of a clinical prediction model in new patients. *J Clin Epidemiol* 2008;61:76-86.

36. Steyerberg EW, Vickers AJ, Cook NR, et al. Assessing the performance of prediction models: a framework for traditional and novel measures. *Epidemiology* 2010;21:128-38.

37. Collins GS, Reitsma JB, Altman DG, et al. Transparent reporting of a multivariable prediction model for individual prognosis or diagnosis (TRIPOD): the TRIPOD statement. *Ann Intern Med* 2015;162:55-63.

38. O'Connor SR, Bleakley CM, Tully MA, McDonough SM. Predicting functional recovery after acute ankle sprain. PLoS One. 2013;8(Suppl 8):e72124.

39. Akacha M, Hutton JS, Lamb SE. Modelling treatment, age- and genderspecific recovery in acute injury studies. In: The University of Warwick Centre for Research in Statistical Methodology. 2010;10:11-12. http://www2.warwick.ac.uk/fac/sci/statistics/crism/research/2010/paper10-09. Accessed 17 Feb 2015.

40. van Middelkoop M, van Rijn RM, Verhaar JA, Koes BW, Bierma-Zeinstra SM. Re-sprains during the first 3 months after initial ankle sprain are related to incomplete recovery: an observational study. J Phys. 2012;58(Suppl 3):181–8.

41. Medina McKeon JM, Bush HM, Reed A, Whittington A, Uhl TL, McKeon PO. Return-to-play probabilities following new versus recurrent ankle sprains in high school athletes. J Sci Med Sport. 2014;17(Suppl 1):23–8.

42. Pourkazemi F, Hiller CE, Raymond J, Nightingale EJ, Refshauge KM. Predictors of chronic ankle instability after an index lateral ankle sprain: a systematic review. J Sci Med Sport / Sports Med Aust. 2014;17(Suppl 6):568–73.

For peer review only - http://bmjopen.bmj.com/site/about/guidelines.xhtml

FIGURE LEGENDS

Figure 1. Decision curve analysis for the baseline and updated (baseline plus 4-weeks predictors) prognostic models.

Figure 2. Calibration plots for the baseline (left) and updated (right) SPRAINED prognostic models, overlaying calibration lines derived from the analyses of 50 imputed datasets.

For peer review only

For peer review only - http://bmjopen.bmj.com/site/about/guidelines.xhtml

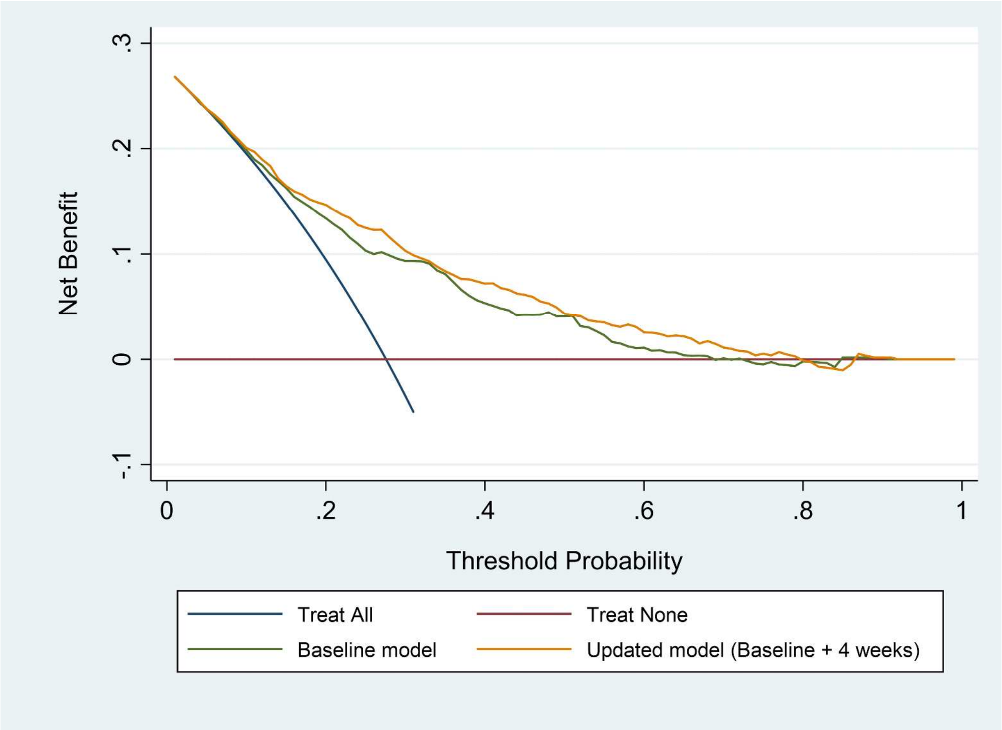

Caption : Figure 1. Decision curve analysis for the baseline and updated (baseline plus 4-weeks predictors) prognostic models.

278x202mm (300 x 300 DPI)

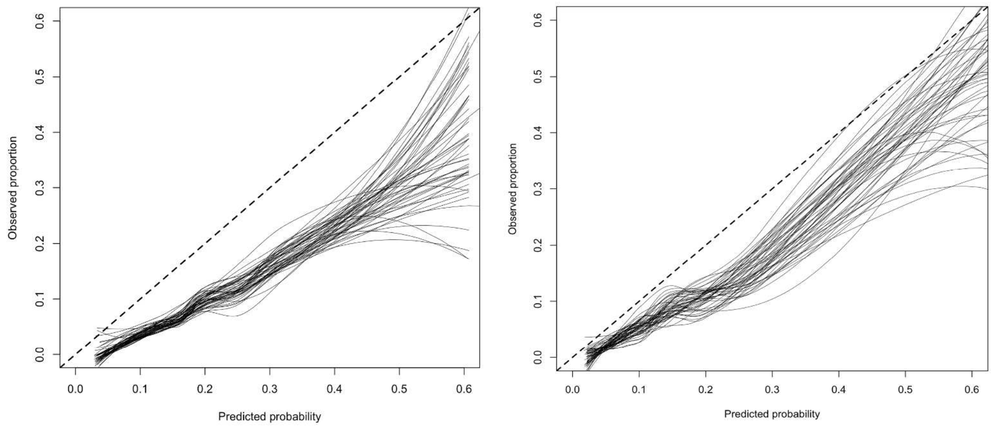

Figure 2. Calibration plots for the baseline (left) and updated (right) SPRAINED prognostic models, overlaying calibration lines derived from the analyses of 50 imputed datasets.

342x152mm (300 x 300 DPI)

For peer review only

Supplemental Table 1. List of candidate predictor variables from the ED presentation.

ED Proforma

1. Date of birth
2. Sex (male / female / no response)
3. Date of ED visit
4. Date of injury
5. Location of pain
6. Anterior Drawer Test (Positive / Painful / Negative / no response)
7. Talar Tilt Test (Positive / Painful / Negative / no response)
8. Proximal Fibular tender (Positive / Painful / Negative / no response)
9. Weight bearing ability (Full / Partial / None / no response)
10. X-ray (yes / no / no response)
11. Crutches (yes / no/ no response)
12. Trial Reason for not entering trial (ankle fracture / other recent fracture / contraindication to intervention / poor skin viability / > 7 days since injury / other)
13. Additional information (if other)
14. Recruiting Centre
15. Date of Trial Clinic
16. Days since injury

For peer review only

Supplemental Table 2. List of candidate predictor variables from the Baseline assessment datasets.

Identifier variables

1. Trial Centre
2. Patient's ID
3. Date of assessment
4. Randomisation Group
5. Treatment Received
6. Calendar code
7. Calendar colour
8. Indicator of Pilot Study phase (I / II / main trial)
9. Response at baseline (yes / no)

Background Information Form

10. Age (years)
11. Sex (male / female)
12. Ethnic Group (White / Black-Caribbean / Black-African / Black-Other / Indian / Pakistani / Bangladeshi / Chinese / Other)
13. Ethnic Group details (if other)
14. First language (English / Other European / Gujarati / Hindi / Punjabi / Urdu / Bengali / Other)
15. First language additional info (if other)
16. Able to answer English questions? (yes / no)
17. Current employment status (full-time / part-time / unemployed)
18. Employment category (paid / unpaid)
19. Hours employed (Less than 10 / 10-25 / 25-40 / More than 40 hours per week)
20. Type of employment (Unskilled manual / skilled manual / unskilled non-manual / skilled non-manual / professional / other / decline d to answer)
21. Description of employment (if professional)
22. Description of employment (if other)
23. Occupation if not employed (retired / not looking for work / unable to work / looking for work / full time student / other)
24. Description of unemployment (if other)
25. Education (CSE / O-Level or GCSE / A-level / degree / higher degree / other)
26. Description of level of education (if other)
27. Time on feet (most of the day / > 4 hours a day / < 4 hours a day / Not much time, mostly sitting)
28. Time driving (most of the day / > 4 hours a day / < 4 hours a day / just to & from work / don't drive)
29. Current medications (since ankle injury / prior to injury / no / no answer)
30. Practice of physical activities (11 questions) (> once a week / < once a week / never)
41. Other physical activity (if other)
42. Height (cm)
43. Weight (kg)
44. Pain before injury (yes/no)
45. When had previous pain (exercise or heavy activities / exercise and daily activities / constantly / other)
46. Description of when had previous pain (if other)
47. Frequency of previous pain (never / monthly / weekly / daily / always)
48. Previous instability (yes / no)
49. Severity of instability (mild / moderate / severe)
50. Frequency of instability (rarely / sometimes / frequently / always)
51. Previous injury (yes / no)
52. 3+ previous injuries (yes / no)
53. Previous injury <1 year ago (yes / no)
54. Recurrent sprain – yes to all 3 above (yes / no)
55. A&E attendance previously (yes / no)
56. How present injury occurred (during sport / at work / at home / outside in public place / other)
57. Description of how present injury occurred
58. Maximum weight bearable (kg)

Baseline questionnaire

59. FAOS components (42 questions)
101. Pain at rest VAS (0-100)
102. Pain bearing weight VAS (0-100)
103. FAOS Baseline symptoms (subscale)
104. FAOS Baseline pain (subscale)
105. FAOS Baseline function ADL (subscale)
106. FAOS Baseline function sport (subscale)
107. FAOS Baseline QoL (subscale)
108. FLP components (13 questions)
121. WORK components (10 questions)
131. FLP score
132. WORK score
133. 1998 SF-12 components (12 questions)
145. 1998 SF-12 physical score
146. 1998 SF-12 mental score
147. Baseline EQ-5D components (5 questions)
152. Baseline EQ-5D score
153. General level of health today (better / same / worse than the past 6 months)
154. VAS health today (0-100)

Note: Imputed scores of validated scales with specific rules for handling missing data imputation (such as FAOS, SF-12 and EQ-5D) are also present in the CAST dataset, but were not described here.

For peer review only - http://bmjopen.bmj.com/site/about/guidelines.xhtml

Supplemental Table 3. Pre-selected baseline and 4-weeks candidate predictor variables.

Type	Variable name	Categories / units	Missing values	
Baseline				
Binary	Gender	Male, female	0	0%
	Previous pain	Yes, No	26	4%
	Recurrent sprain	Yes, No	12	2%
Categorical	Employment status	No, Part time, Full time	0	0%
	Education	CSE, GCSE, A Level, Degree, Higher Degree	20	3%
	Anterior Drawer Test	Positive, Painful, Negative, No response	396	68%
	Talar Tilt Test	Positive, Painful, Negative, No response	403	69%
	Proximal Fibular tender ligament test	Positive, Painful, Negative, No response	378	65%
	Able to bear weight	Full / partial / none	322	55%
	Treatment group	Tubular bandage, Below knee cast, Aircast brace, Bledsoe boot	0	0%
	Leisure time physical activity	None, <1 weekly, >1 weekly	7	1%
	Walking 2 miles or more	None, <1 weekly, >1 weekly	24	4%
	Previous instability	None, Mild, Moderate, Severe	27	5%
	Previous instability frequency	Never, Rarely, Sometimes, Frequently, Always	29	5%
	Injury presentation	During sport, at work, at home, outside in public	34	6%
	Ankle/foot Swelling[1]	Never, Rarely, Sometimes, Often, Always	18	3%
	Ankle/foot Grinding/clicking[1]	Never, Rarely, Sometimes, Often, Always	18	3%
	Ankle/foot catching/locking[1]	Never, Rarely, Sometimes, Often, Always	18	3%
	Ankle ROM plantar flexion[1]	Never, Rarely, Sometimes, Often, Always	18	3%
	Ankle ROM plantar dorsiflexion[1]	Never, Rarely, Sometimes, Often, Always	18	3%
	Pain at night (on bed) [1]	None, Mild, Moderate, Severe, Extreme	18	3%
	Difficulty with squatting[1]	None, Mild, Moderate, Severe, Extreme	29	5%
	Difficulty with running[1]	None, Mild, Moderate, Severe, Extreme	31	5%
	Difficulty with jumping[1]	None, Mild, Moderate, Severe, Extreme	31	5%
	Difficulty with twisting/pivoting[1]	None, Mild, Moderate, Severe, Extreme	26	4%
Continuous	Time since injury	0-7 days	321	55%
	Age	Years[2]	0	0%
	Body mass index[3]	kg/m^2	19	3%
	Maximum weight bearable	kg	5	1%
	Pain when resting	Visual analogue scale (0-100)	4	1%
	Pain when bearing weight	Visual analogue scale (0-100)	9	2%
	SF-12 mental component	Score (0-100)	5	1%
4-weeks				
Binary	Repeat injury to the same ankle	Yes, No	118	20%
	Returned to ED due to repeated injury	Yes, No	120	21%
Categorical	Returned to usual sports/activities	No, partially, fully	121	21%
	Ankle/foot Swelling	Never, Rarely, Sometimes, Often, Always	102	17%
	Ankle/foot Grinding/clicking	Never, Rarely, Sometimes, Often, Always	102	17%
	Ankle/foot catching/locking	Never, Rarely, Sometimes, Often, Always	103	18%
	Ankle ROM plantar flexion	Never, Rarely, Sometimes, Often, Always	102	17%
	Ankle ROM plantar dorsiflexion	Never, Rarely, Sometimes, Often, Always	102	17%
	Pain at night	None, Mild, Moderate, Severe, Extreme	101	17%
	Difficulty with squatting	None, Mild, Moderate, Severe, Extreme	101	17%
	Difficulty with running	None, Mild, Moderate, Severe, Extreme	135	22%
	Difficulty with jumping	None, Mild, Moderate, Severe, Extreme	137	23%
	Difficulty with twisting/pivoting	None, Mild, Moderate, Severe, Extreme	131	22%
Continuous	Pain at weight bearing	0-100	196	34%

For peer review only

Supplemental Table 4. Reasons for exclusion of baseline and 4-weeks candidate predictors before statistical modelling.

Predictor	Reason for exclusion
Baseline	
Anterior Drawer Test	≥ 60% missing values, consensus agreement
Talar Tilt Test	≥ 60% missing values, consensus agreement
Proximal Fibular tender ligament test	≥ 60% missing values, consensus agreement
Ankle/foot Swelling	One or more cells with too few cases when cross-tabulated with the outcomes, regardless of re-categorisation
Difficulty with running	Highly correlated with "Difficulty with jumping" and "Difficulty with twisting/pivoting". Composite variable used instead.
Difficulty with jumping	Highly correlated with "Difficulty with running" and "Difficulty with twisting/pivoting". Composite variable used instead.
Difficulty with twisting/pivoting	Highly correlated with "Difficulty with running" and "Difficulty with jumping". Composite variable used instead.
Previous instability	Highly correlated with "Previous instability frequency"
Previous instability frequency	"Perfect prediction" during missing data multiple imputation
Difficulty with running/jumping/twisting	"Perfect prediction" during missing data multiple imputation
4 weeks follow-up	
Returned to ED due to repeated injury	One or more cells with too few cases when cross tabulated with the outcomes
Difficulty with running	Highly correlated with "Difficulty with jumping" and "Difficulty with twisting/pivoting". Composite variable used instead.
Difficulty with jumping	Highly correlated with "Difficulty with running" and "Difficulty with twisting/pivoting". Composite variable used instead.
Difficulty with twisting/pivoting	Highly correlated with "Difficulty with running" and "Difficulty with jumping". Composite variable used instead.
Ankle ROM plantar dorsiflexion	"Perfect prediction" during missing data multiple imputation

Note: Perfect prediction occurs whenever there is a level of a categorical explanatory variable for which the observed values of the outcome are all 1 (or all 0); it is often resolved by discarding the observations corresponding to offending covariate patterns or the independent variables perfectly predicting outcomes during estimation.

For peer review only

Supplemental Table 5. SPRAINED baseline and updated prognostic models' equations.

Baseline SPRAINED prognostic model:

Y = -3.68 + (0.02 × age) + (0.02 × BMI) + (0.01 × pain when resting) + (0.01 × pain when bearing weight) + (0.61 if days from injury to assessment > 2) – (0.56 if able to bear any weight on the injured ankle) + (0.84 if the injury is a recurrent sprain)

Updated SPRAINED prognostic model (baseline + 4-weeks predictors):

Y = -4.4 + (0.01 × age) + (0.02 × BMI) + (0.01 × pain when resting) + (0.01 × pain when bearing weight) + (0.59 if days from injury to assessment > 2) – (0.68 if able to bear any weight on the injured ankle) + (0.99 if the injury is a recurrent sprain) + (0.02 × pain when bearing weight 4 weeks after injury)

The log odds (*Y*) estimated with these equations must be converted into a probability (*P*), which can be done by applying the following equation:

$$P = 1/(1 + \exp(-Y))$$

For peer review only

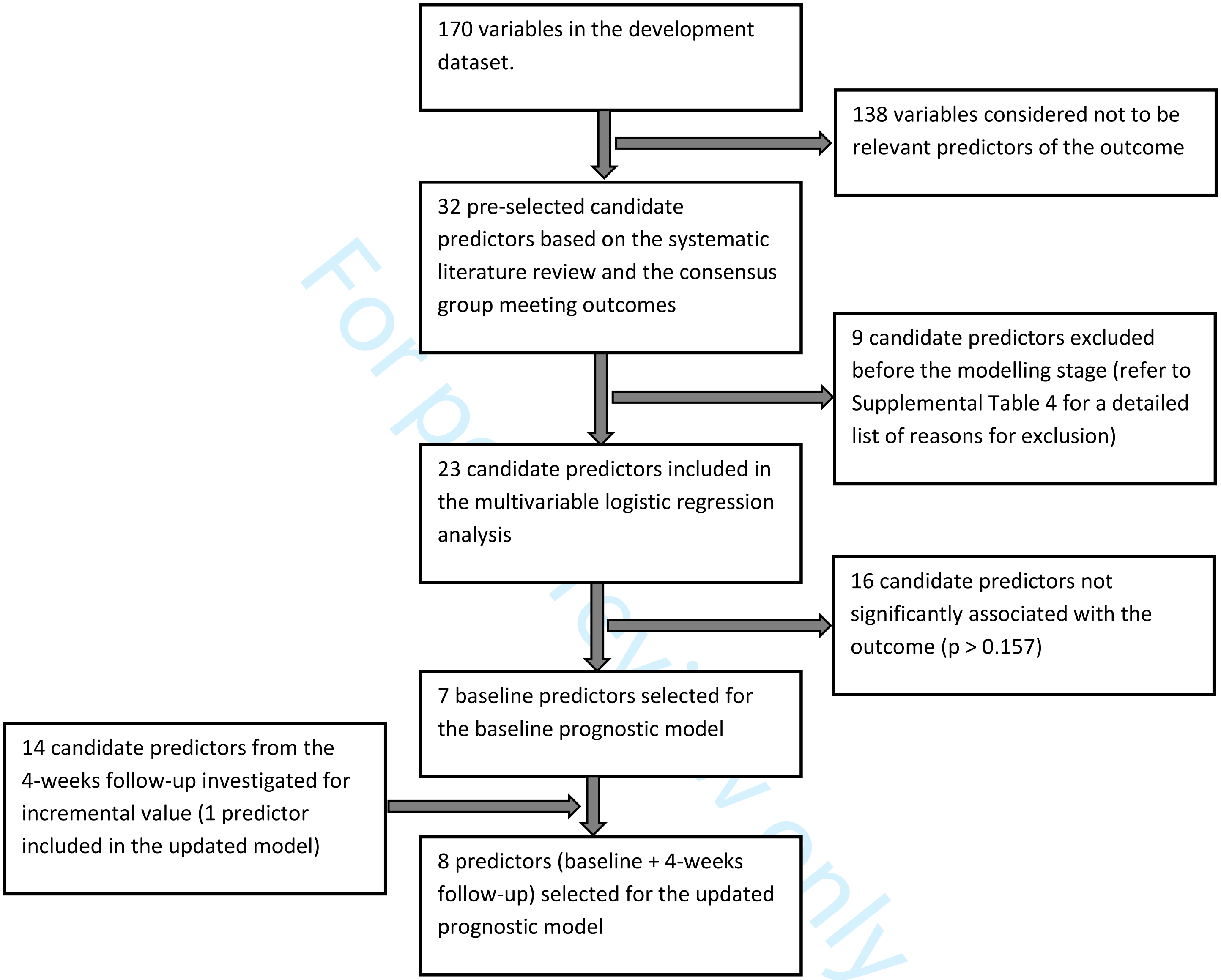

Supplemental Figure 1. Summary of the selection process for predictors included in the SPRAINED prognostic model.

For peer review only - http://bmjopen.bmj.com/site/about/guidelines.xhtml

TRIPOD

TRIPOD Checklist: Prediction Model Development and Validation

Section/Topic	Item		Checklist Item	Page
**Title and abstract**				
Title	1	D;V	Identify the study as developing and/or validating a multivariable prediction model, the target population, and the outcome to be predicted.	1
Abstract	2	D;V	Provide a summary of objectives, study design, setting, participants, sample size, predictors, outcome, statistical analysis, results, and conclusions.	2
**Introduction**				
Background and objectives	3a	D;V	Explain the medical context (including whether diagnostic or prognostic) and rationale for developing or validating the multivariable prediction model, including references to existing models.	4,5
	3b	D;V	Specify the objectives, including whether the study describes the development or validation of the model or both.	5
**Methods**				
Source of data	4a	D;V	Describe the study design or source of data (e.g., randomized trial, cohort, or registry data), separately for the development and validation data sets, if applicable.	5,6
	4b	D;V	Specify the key study dates, including start of accrual; end of accrual; and, if applicable, end of follow-up.	5
Participants	5a	D;V	Specify key elements of the study setting (e.g., primary care, secondary care, general population) including number and location of centres.	5
	5b	D;V	Describe eligibility criteria for participants.	5
	5c	D;V	Give details of treatments received, if relevant.	5
Outcome	6a	D;V	Clearly define the outcome that is predicted by the prediction model, including how and when assessed.	6
	6b	D;V	Report any actions to blind assessment of the outcome to be predicted.	Not done
Predictors	7a	D;V	Clearly define all predictors used in developing or validating the multivariable prediction model, including how and when they were measured.	6,8
	7b	D;V	Report any actions to blind assessment of predictors for the outcome and other predictors.	Not done
Sample size	8	D;V	Explain how the study size was arrived at.	8
Missing data	9	D;V	Describe how missing data were handled (e.g., complete-case analysis, single imputation, multiple imputation) with details of any imputation method.	7 8
Statistical analysis methods	10a	D	Describe how predictors were handled in the analyses.	7,8
	10b	D	Specify type of model, all model-building procedures (including any predictor selection), and method for internal validation.	7,8
	10c	V	For validation, describe how the predictions were calculated.	8
	10d	D;V	Specify all measures used to assess model performance and, if relevant, to compare multiple models.	8,9
	10e	V	Describe any model updating (e.g., recalibration) arising from the validation, if done.	8
Risk groups	11	D;V	Provide details on how risk groups were created, if done.	Not done
Development vs. validation	12	V	For validation, identify any differences from the development data in setting, eligibility criteria, outcome, and predictors.	5,6
**Results**				
Participants	13a	D;V	Describe the flow of participants through the study, including the number of participants with and without the outcome and, if applicable, a summary of the follow-up time. A diagram may be helpful.	9,10
	13b	D;V	Describe the characteristics of the participants (basic demographics, clinical features, available predictors), including the number of participants with missing data for predictors and outcome.	9,10
	13c	V	For validation, show a comparison with the development data of the distribution of important variables (demographics, predictors and outcome).	9,10
Model development	14a	D	Specify the number of participants and outcome events in each analysis.	8,9
	14b	D	If done, report the unadjusted association between each candidate predictor and outcome.	Not done
Model specification	15a	D	Present the full prediction model to allow predictions for individuals (i.e., all regression coefficients, and model intercept or baseline survival at a given time point).	Table 3
	15b	D	Explain how to the use the prediction model.	Table S5
Model performance	16	D;V	Report performance measures (with CIs) for the prediction model.	13
Model-updating	17	V	If done, report the results from any model updating (i.e., model specification, model performance).	Not done
**Discussion**				
Limitations	18	D;V	Discuss any limitations of the study (such as nonrepresentative sample, few events per predictor, missing data).	16
Interpretation	19a	V	For validation, discuss the results with reference to performance in the development data, and any other validation data.	15,16
	19b	D;V	Give an overall interpretation of the results, considering objectives, limitations, results from similar studies, and other relevant evidence.	14
Implications	20	D;V	Discuss the potential clinical use of the model and implications for future research.	16,17
**Other information**				
Supplementary information	21	D;V	Provide information about the availability of supplementary resources, such as study protocol, Web calculator, and data sets.	17
Funding	22	D;V	Give the source of funding and the role of the funders for the present study.	17,18

For peer review only - http://bmjopen.bmj.com/site/about/guidelines.xhtml

TRIPOD Checklist: Prediction Model Development and Validation

*Items relevant only to the development of a prediction model are denoted by D, items relating solely to a validation of a prediction model are denoted by V, and items relating to both are denoted D;V. We recommend using the TRIPOD Checklist in conjunction with the TRIPOD Explanation and Elaboration document.

For peer review only
